# Metabolic flexibilities and vulnerabilities in the pentose phosphate pathway of the zoonotic pathogen *Toxoplasma gondii*

**DOI:** 10.1371/journal.ppat.1010864

**Published:** 2022-09-19

**Authors:** Ningbo Xia, Xuefang Guo, Qinghong Guo, Nishith Gupta, Nuo Ji, Bang Shen, Lihua Xiao, Yaoyu Feng

**Affiliations:** 1 Guangdong Laboratory for Lingnan Modern Agriculture, College of Veterinary Medicine, South China Agricultural University, Guangzhou, China; 2 Department of Biological Sciences, Birla Institute of Technology and Science, Pilani (Hyderabad Campus), Hyderabad, India; 3 Department of Molecular Parasitology, Faculty of Life Sciences, Humboldt University, Berlin, Germany; 4 State Key Laboratory of Agricultural Microbiology, College of Veterinary Medicine, Huazhong Agricultural University, Wuhan, China; University of Geneva, SWITZERLAND

## Abstract

Metabolic pathways underpin the growth and virulence of intracellular parasites and are therefore promising antiparasitic targets. The pentose phosphate pathway (PPP) is vital in most organisms, providing a reduced form of nicotinamide adenine dinucleotide phosphate (NADPH) and ribose sugar for nucleotide synthesis; however, it has not yet been studied in *Toxoplasma gondii*, a widespread intracellular pathogen and a model protozoan organism. Herein, we show that *T*. *gondii* has a functional PPP distributed in the cytoplasm and nucleus of its acutely-infectious tachyzoite stage. We produced eight parasite mutants disrupting seven enzymes of the PPP in *T*. *gondii*. Our data show that of the seven PPP proteins, the two glucose-6-phosphate dehydrogenases (*Tg*G6PDH1, *Tg*G6PDH2), one of the two 6-phosphogluconate dehydrogenases (*Tg*6PGDH1), ribulose-5-phosphate epimerase (*Tg*RuPE) and transaldolase (*Tg*TAL) are dispensable *in vitro* as well as *in vivo*, disclosing substantial metabolic plasticity in *T*. *gondii*. Among these, *Tg*G6PDH2 plays a vital role in defense against oxidative stress by the pathogen. Further, we show that *Tg*6PGDH2 and ribulose-5-phosphate isomerase (*Tg*RPI) are critical for tachyzoite growth. The depletion of *Tg*RPI impairs the flux of glucose in central carbon pathways, and causes decreased expression of ribosomal, microneme and rhoptry proteins. In summary, our results demonstrate the physiological need of the PPP in *T*. *gondii* while unraveling metabolic flexibility and antiparasitic targets.

## Author summary

Metabolic pathways are intimately associated with the survival and replication of parasitic *Toxoplasma gondii* and thus represent potential targets for antiparasitic strategies. Herein, we focused on the pentose phosphate pathway (PPP) in *T*. *gondii* and examined its roles in supporting the growth of this ubiquitous pathogen. We found that *Tg*G6PDH1 and *Tg*G6PDH2 were needed to defend oxidative stress but not for pentose synthesis. We revealed that inactivation of the *Tg*6PGDH2 and *Tg*RPI severely impaired the asexual reproduction of tachyzoites. We also highlighted the remarkable metabolic plasticity in tachyzoites that enables them to acquire some of the PPP intermediates from multiple routes. This study provides significant insights into the carbon metabolism properties of *Toxoplasma* parasites, opening avenues for targeting this pathway to develop therapeutic interventions against toxoplasmosis.

## Introduction

*Toxoplasma gondii*, an obligate intracellular parasite capable of infecting virtually all nucleated cells in diverse organisms, is the causative agent of zoonotic toxoplasmosis [1]. It has a complex life cycle, including both asexual and sexual stages [2]. The asexual phase of the parasite can occur in several intermediate hosts, such as humans, pigs, and sheep, whereas sexual development is restricted to the feline intestine [1–3]. The parasite is known to flexibly reprogram its metabolism for surviving and proliferating in diverse host cell types and nutritional milieus [4–13].

*Toxoplasma gondii* utilizes glucose as a primary carbon source to support the metabolic demands during its asexual reproduction in mammalian cells [5,14–16]. Glucose is imported by facilitative glucose transporter (*Tg*GT1) into the parasite cytoplasm where it is metabolized by glycolysis and/or pentose phosphate pathway (PPP), branching at glucose 6-phosphate (G6P) [5,17,18]. Several studies have suggested that the central carbon metabolism is critical for optimal asexual growth and metabolic flexibility in *Toxoplasma* [6,16,18,19]. Although PPP is also a key route of glucose catabolism, its contribution to parasite metabolism and pathogenesis remains largely unexplored.

The parasite encodes all enzymes of the oxidative and non-oxidative branches of PPP [17,20] (www.ToxoDB.org). The oxidative branch consists of two glucose-6-phosphate dehydrogenases (*Tg*G6PDH1 and *Tg*G6PDH2) and two 6-phosphogluconate dehydrogenases (*Tg*6PGDH1 and *Tg*6PGDH2) enzymes [17]. *Tg*G6PDHs catalyze G6P to 6-phosphogluconolactone (6PGL) [17]. *Tg*6PGDH catalyzes 6PG to ribulose-5-phosphate (Ru5P) [17]. Both reactions generate NADPH which is required as a reducing equivalent by other metabolic pathways including fatty acid synthesis [6,21]. The resulting Ru5P is then catalyzed to ribose 5-phosphate (R5P) and xylulose-5-phosphate (Xu5P) by ribose 5-phosphate isomerase (*Tg*RPI) and ribulose 5-phosphate 3-epimerase (*Tg*RuPE), respectively [17,22]. The non-oxidative branch of PPP in *T*. *gondii* comprises a set of reactions in which R5P and X5P are converted into fructose 6-phosphate (F6P) and glyceraldehyde 3-phosphate (GA3P) by transketolase (*Tg*TKT) and transaldolase (*Tg*TAL) [17]. The F6P and GA3P can then be utilized *via* glycolysis. Notably, however, the physiological significance and metabolic contribution of these enzymes in *T*. *gondii* have not yet been investigated.

Herein, we performed a functional analysis of the PPP metabolism in *T*. *gondii*. We generated eight mutant strains, disrupting seven enzymes, and analyzed their phenotypic and metabolic features. This study suggests that the pentose phosphate pathway supports the parasite metabolism, growth and virulence. Our work also emphasizes the metabolic plasticity of carbon metabolism during the lytic cycle of *T*. *gondii*.

## Results

### The pentose phosphate pathway localizes in the cytoplasm and nucleus of *Toxoplasma gondii*

**Fig 1:**
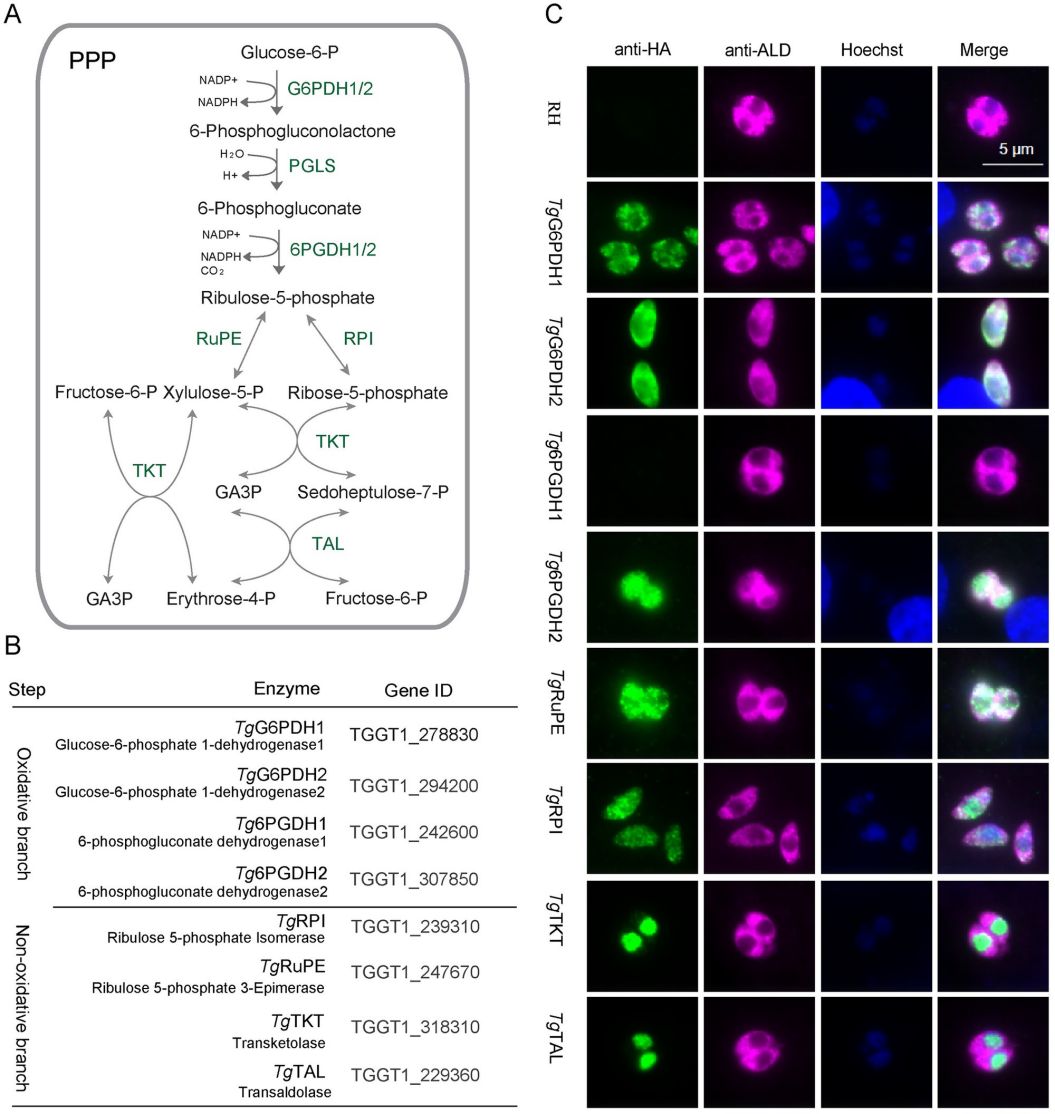
*Toxoplasma gondii* encodes its pentose phosphate pathway within 2 subcellular locations. **(A)**, Schematic representation of the pentose phosphate pathway in *Toxoplasma* parasites. **(B)**, Gene ID of the PPP genes. **(C)**, Representative images of immunofluorescence assay. Different enzymes of PPP tagged at the C terminus with smHA epitope by CRISPR-Cas9-mediated homologous recombination in the RH*Δku80* strain. Subsequently, the parasites of testing strains were fixed, permeabilized and stained with mouse anti-HA and rabbit anti-*Tg*ALD, which were detected by Alexa 488- and Alexa 594-conjugated secondary antibodies, respectively. *Tg*ALD and Hoechst were used as the cytoplasm and cell nucleus markers, respectively. Bar = 5 μm.

The parasite genome encodes eight genes predicted to be the enzymes of PPP (Fig 1A), namely *TgG6PDH1*, *TgG6PDH2*, *Tg6PGDH1*, *Tg6PGDH2*, *TgRPI*, *TgRuPE*, *TgTKT*, and *TgTAL* (Fig 1B). To examine whether all proteins of the pentose phosphate pathway are expressed in the acute (tachyzoite) stage of *T*. *gondii*, we performed their 3’-genomic tagging with an HA epitope using CRISPR/Cas9 mediated site-specific integration in the RH*Δku80* strain. Immunofluorescent staining of the HA-tagged proteins revealed that *Tg*G6PDH1, *Tg*G6PDH2, *Tg*6PGDH2, *Tg*RuPE and *Tg*RPI were expressed in the tachyzoite cytoplasm, as shown by co-localization with a cytoplasmic marker, *Tg*ALD (Fig 1C) [23]. Especially, to further confirm the cytoplasm localization of the *Tg*6PGDH2 and *Tg*RPI on the level of the native protein, we produced polyclonal antisera against *Tg*6PGDH2 and *Tg*RPI which had been recombinantly expressed in *E*. *coli* BL21 (S1A, S1B and S2A and S2B Figs). Immunofluorescent staining using these antisera showed that the native *Tg*6PGDH2 and *Tg*RPI were expressed in the cytoplasm of *T*. *gondii* (S1C and S2C Figs). Unexpectedly, *Tg*TKT and *Tg*TAL co-localized with the nucleus marker Hoechst (Fig 1C), and *Tg*6PGDH1 is not expressed in *Toxoplasma* tachyzoites, as confirmed by immunofluorescent staining (Fig 1C).

### *Tg*G6PDH1/2, *Tg*6PGDH1, *Tg*RuPE and *Tg*TAL are dispensable for the lytic cycle

**Fig 2:**
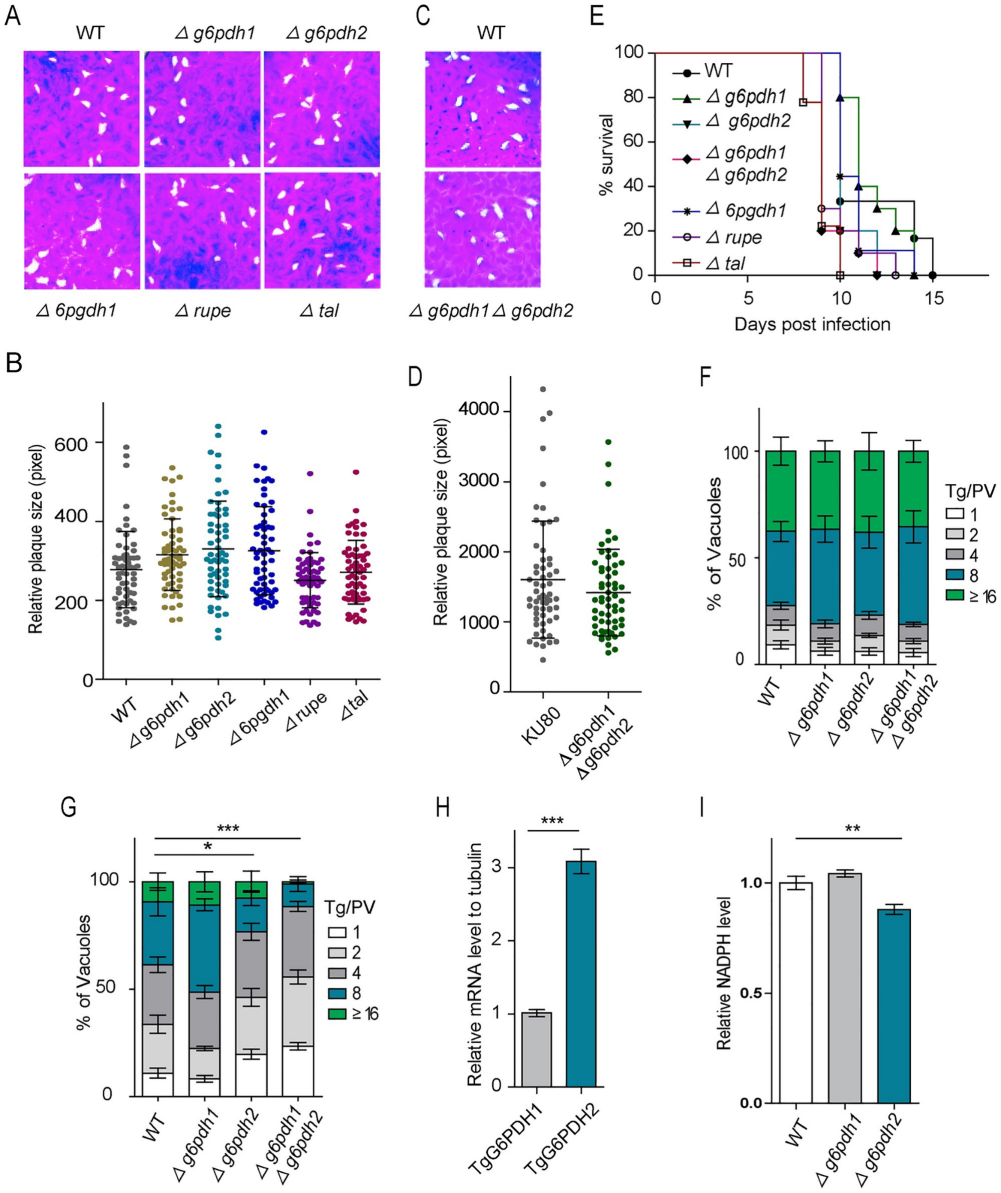
*Tg*G6PDH1, *Tg*G6PDH2, *Tg*6PGDH1, *Tg*RuPE and *Tg*TAL are dispensable for parasite growth and virulence, while *Tg*G6PDHs are required for defending against oxidative stress. **(A)**, Plaque assay comparing the growth of *Δg6pdh1*, *Δg6pdh2*, *Δ6pgdh1*, *Δrupe* and *Δtal* tachyzoites *in vitro* to that of wild-type strain RH*Δku80*. **(B)**, Relative sizes (pixel size calculated by photoshop) of plaques. Means ± SD of >60 plaques (n = 3). **(C)**, Representative Plaques image showing the comparative growth of *Δg6pdh1Δg6pdh2* and RH*Δku80* strains. **(D)**, Graph presentation of plaque sizes of *Δg6pdh1Δg6pdh2* and RH*Δku80* strains. Means ± SD of >60 plaques (n = 3). **(E)**, Virulence tests of indicated strains in ICR mice (100 parasites per mouse, 10 mice for each strain). **(F)**, Intracellular replication assay comparing parasite proliferation under standard culture conditions. Freshly egressed tachyzoites of RH*Δku80*, *Δg6pdh1*, *Δg6pdh2* and *Δg6pdh1Δg6pdh2* parasites were allowed to infect HFF monolayers for 1 h, and invaded parasites were cultured at 37°C with 5% CO_2_ for another 24 h. Percentiles of the parasitophorous vacuole (PV) containing 1, 2, 4, 8, 16, or more parasites were determined and plotted. Means ± SEM from three independent experiments (n = 3). **(G)**, Freshly egressed tachyzoites of indicated strains were pre-treated with 500 μM H_2_O_2_ medium for 3 h, and then subjected to intracellular replication assay (without H_2_O_2_) for 24 h. Means ± SEM from three independent experiments was graphed. Two-way ANOVA, *, *p <0* .*05*, ***, *P < 0*.*001*. **(H)**, RH*Δku80* tachyzoites were purified, total RNA was extracted and reversed into cDNA. Transcript levels for *Tg*G6PDH1 and *Tg*G6PDH2 in each sample were analyzed by quantitative real-time PCR, using β-tubulin as an internal reference. Means ± SEM of four independent experiments (n = 4). Student’s t-test, ***, *P<0*.*001*. **(I)**, Parasites (approximately 3×10^7^) were purified by 3 μm membrane filtration, washed with cold PBS and extracted with extraction buffer. Relative NADPH levels in RH*Δku80* (WT), *Δg6pdh1* and *Δg6pdh2* were determined by the NADPH Assay kit. Means ± SEM from three independent experiments (n = 3). **, *P<0*.*01*; one-way ANOVA.

To evaluate the physiological significance of each PPP gene to parasite fitness, we attempted to delete the *TgG6PDH1*, *TgG6PDH2*, *Tg6PGDH1*, *Tg6PGDH2*, *TgRPI*, *TgRuPE*, *TgTKT* and *TgTAL* genes through CRISPR/Cas9-mediated homologous gene replacement in the RH*Δku80* strain (S3A Fig). The direct knockout strains of *Δg6pdh1*, *Δg6pdh2*, *Δ6pgdh1*, *Δrupe* and *Δtal* were obtained after pyrimethamine selection, as confirmed by PCR and semi-quantitative RT-PCR screening of the clonal mutants (S3B–S3J Fig). However, no viable *Δ6pgdh2*, *Δrpi* and *Δtkt* mutants could be generated, suggesting a critical function of the corresponding proteins in tachyzoites. The number and size of plaques formed by the *Δg6pdh1*, *Δg6pdh2*, *Δ6pgdh1*, *Δrupe* and *Δtal* mutants were similar to that of the parental RH*Δku80* strain (Fig 2A and 2B), indicating that *Tg*G6PDH1, *Tg*G6PDH2, *Tg*6PGDH1, *Tg*RuPE and *Tg*TAL enzymes are not required during the lytic cycle.

*Toxoplasma* harbors two *Tg*G6PDH enzymes. We tested their functional redundancy by generating a *Δg6pdh1Δg6pdh2* double mutant, in which the *TgG6PDH1* gene was replaced by the CAT selection marker in the *Δg6pdh2* mutant (S4A Fig). The diagnostic PCRs and semi-quantitative RT-PCR confirmed the deletion of the *TgG6PDH1* locus in the *Δg6pdh2* strain (S4B and S4C Fig). The absence of both genes in the double mutant was endorsed by whole-genome sequencing (S4D and S4E Fig). Consistently, the RNA sequencing revealed the downregulation of *TgG6PDH1* and *TgG6PDH2* in the *Δg6pdh1Δg6pdh2* strain (S4F Fig). Remarkably, the double-deletion strain showed no apparent phenotypic defects *in vitro* as examined by routine culture, plaque and replication assays (Fig 2C, 2D and 2F), which suggested that tachyzoites can indeed survive with the loss of the two *Tg*G6PDH isoforms.

To test the consequences of gene deletion on the parasite virulence, ICR mice were intraperitoneally injected with the *Δg6pdh1*, *Δg6pdh2*, *Δg6pdh1Δg6pdh2*, *Δ6pgdh1*, *Δrupe* and *Δtal* strains, and their survival was monitored for 30 days. Interestingly, none of them showed an attenuated virulence (Fig 2E), suggesting that *Tg*G6PDH1/2, *Tg*6PGDH1, *Tg*RuPE and *Tg*TAL are expendable for *in vivo* metabolism of *T*. *gondii* tachyzoites and thereby not required for the parasite virulence in a mouse model.

### *Tg*G6PDH1/2 are needed to defend oxidative stress

*Tg*G6PDH enzymes are predicted to catalyze the first step in the oxidative pentose phosphate pathway and provide pentose sugars for the nucleotide synthesis [17]. To assess the functional contribution of *Tg*G6PDH, we performed [^13^C_6_]-glucose labeling of extracellular tachyzoites and estimated carbon flux into PPP, glycolysis and TCA cycle intermediates. We found that the *Δg6pdh1Δg6pdh2* mutant showed increased ^13^C-labeling of glycolytic and TCA cycle intermediates, such as fructose-1,6-bisphosphate, 3-phosphoglyceric acid, 2-phosphoglyceric acid, lactate, fumarate and malate (S5 Fig). Generally, no significant difference was observed in the PPP metabolites. Because NADPH produced by the G6PDH enzyme is thought to be a crucial reductive power to defend against oxidative stress [24–26], we also evaluated the growth of RH*Δku80*, *Δg6pdh1*, *Δg6pdh2* and *Δg6pdh1Δg6pdh2* mutants after pre-treatment with 500 μM H_2_O_2_ for 3 h. The RH*Δku80* and *Δg6pdh1* strains exhibited similar replication rates, as determined by the numeration of tachyzoites in the parasitophorous vacuoles. Notably, although the H_2_O_2_ also affected the proliferation of wild-type parasites, the pre-treated *Δg6pdh2* and pre-treated *Δg6pdh1Δg6pdh2* parasites showed a notable defect compared to the parental strain (Fig 2G), indicating that *Tg*G6PDH2 may be involved in oxidative stress response via NADPH synthesis. Therefore, our further work analyzed the expression differences of *Tg*G6PDH1 and *Tg*G6PDH2, and found that the expression of *Tg*G6PDH2 was significantly higher than that of *Tg*G6PDH1 in *T*. *gondii* tachyzoites (Fig 2H). Subsequently, we measured the abundance of NADPH in the mutants and found that the deletion of *TgG6PDH2* significantly affected NADPH production (Fig 2I). Together, these results suggested that *Tg*G6PDH2 plays a function in maintaining the cytosolic NADP^+^/NADPH balance and thus plays a vital physiological role in the anti-oxidant response of tachyzoites.

### *Tg*6PGDH2 is critical for the asexual reproduction of tachyzoites

**Fig 3:**
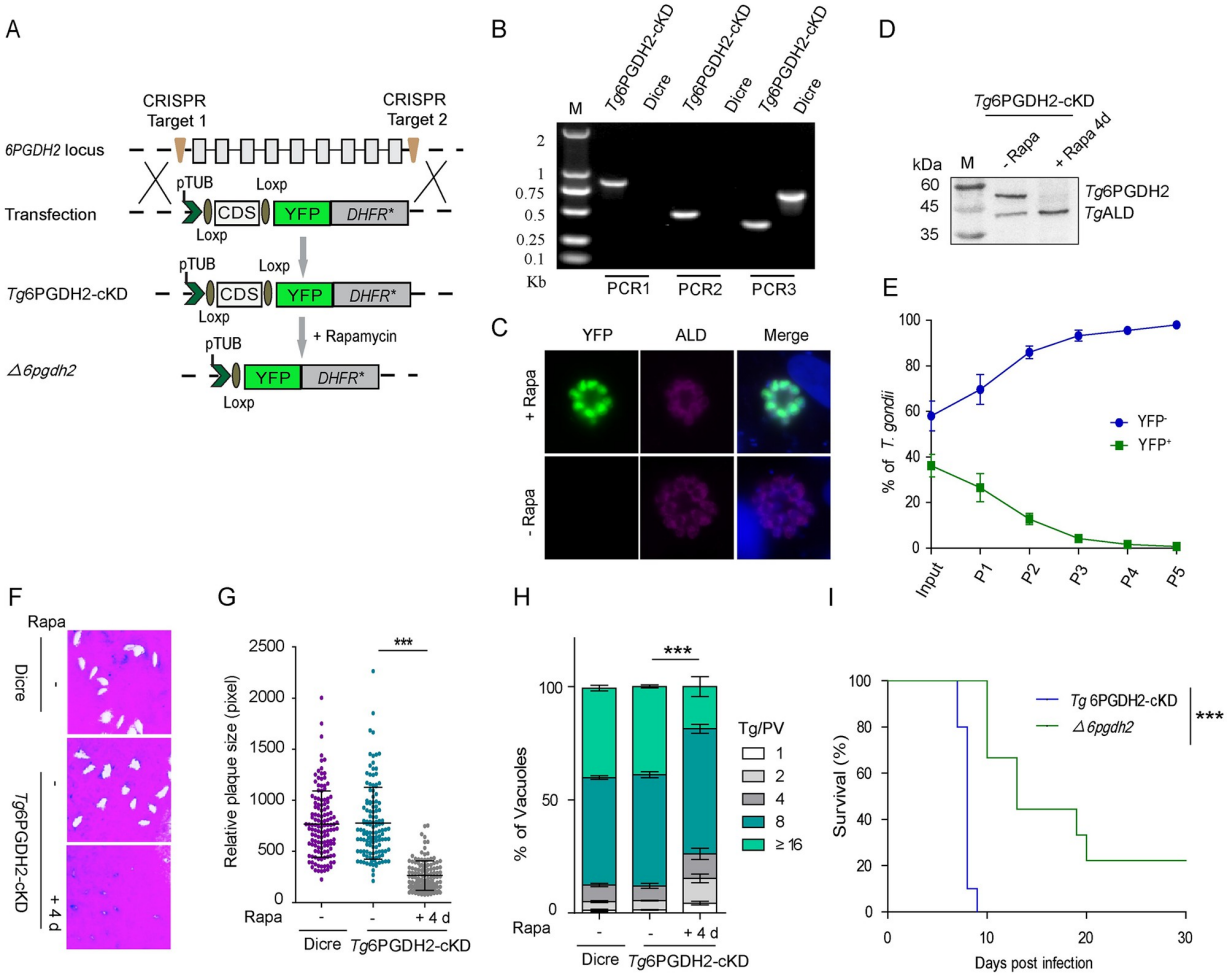
*Tg*6PGDH2 is important for parasite survival. **(A)**, Diagram showing strategy to construct the conditional knockout strain *Tg*6PGDH2-cKD, using the LoxP- Cre system in the DiCre strain. **(B)**, Diagnostic PCRs on a representative *Tg*6PGDH2-cKD clone. PCR1 and PCR2 examined the integration of homology templates at the 5^’^ and 3^’^ end of *Tg6PGDH2*, whereas PCR3 confirmed the deletion of the endogenous *Tg6PGDH2* locus. **(C)**, Immunofluorescence staining for *Tg*ALD and YFP expression in *Tg*6PGDH2-cKD parasites treated with rapamycin for 2 d. **(D)**, Western blotting for checking the expression of *Tg*6PGDH2 in *Tg*6PGDH2-cKD parasites treated with or without rapamycin for 4 days. *Tg*ALD was included as a loading control. **(E)**, Competition assay comparing the growth of *Tg*6PGDH2-cKD parasites treated with rapamycin for 24 h to that of untreated parasites. **(F)**, Plaque assay showing the defect in the *Tg*6PGDH2-cKD parasites after 4 days of rapamycin treatment. **(G)**, *Tg*6PGDH2-cKD parasites pretreated for 4 days formed smaller plaques than their parental strain, and untreated *Tg*6PGDH2-cKD parasites formed plaques similarly to DiCre. **(H)**, Intracellular replication assay comparing parasite growth *in vitro*. *Tg*6PGDH2-cKD parasites were treated with rapamycin for 4 days, and then they were allowed to infect fresh HFF cells and grown for 24 h, subsequently, the number of parasites in each PV was checked by IFA. Results are means ± SEM for n = 3 independent experiments. **(I)**, Survival curves of mice infected with tachyzoites of indicated strains. *Tg*6PGDH2-cKD and *Δ6pgdh2* mutants were used to infect ICR mice (100 parasites/mouse, n = 10 mice for each strain) by intraperitoneal injection, and the survival of mice was followed for 30 days. *, *p <0* .*05*, Gehan–Breslow–Wilcoxon tests.

Our further work focused on *Tg*6PGDH2 that enables decarboxylation of 6PG into Ru5P with concomitant reduction of NADP in tachyzoite cytoplasm. We constructed a rapamycin-inducible *Tg*6PGDH2 mutant using the DiCre system because a direct knockout by CRISPR/Cas9-mediated homologous gene replacement could not be generated. The conditional knockdown mutant was generated *via* pyrimethamine selection, as shown in the scheme (Fig 3A) and confirmed by diagnostic PCR for the intended replacement of the *Tg6PGDH2* gene by pTUB-loxp-*Tg*6PGDH2-loxp-YFP-DHFR* (*Tg*6PGDH2-cKD mutant in Fig 3B). Rapamycin-induced knockout of *Tg*6PGDH2 was confirmed by the appearance of YFP signal in indirect immunofluorescence assays (IFA) (Fig 3C) and further identified by Western blotting (Fig 3D). We cultured the *Tg*6PGDH2-cKD strain with or without rapamycin for 1 day or 4 days, then performed competition, plaque and replication assays under standard culture condition. A knockout of *Tg*6PGDH2 after rapamycin treatment led to significant phenotypic defects *in vitro* as seen by competition assay (Fig 3E), plaque size (Fig 3F and 3G) and vacuole size distribution (Fig 3H). To further check the importance of *Tg*6PGDH2 *in vivo*, a *Tg*6PGDH2-knockout (*Δ6pgdh2*) clonal mutant was produced from rapamycin-treated *Tg*6PGDH2-cKD strain and ascertained by diagnostic PCR (S6A Fig) and Western blotting (S6B Fig). The *Δ6pgdh2* parasites were viable despite their growth significantly slowed down. Subsequently, purified *Δ6pgdh2* tachyzoites were used to infect ICR mice by intraperitoneal injection, and the survival of mice was monitored for 30 days. Typical results of such virulence tests indicated that *Tg6PGDH2* deletion led to attenuated virulence (Fig 3I).

To confirm the specificity of the observed growth defect, we complemented the *Tg*6PGDH2-cKD strain with a *Tg*6PGDH2-expressing cassette into the *UPRT* locus (S7A Fig). The complemented strain (comp*Tg*6PGDH2) was confirmed by diagnostic PCRs (S7B Fig). As expected, the growth defect was fully restored in the comp*Tg*6PGDH2 strain as shown by plaque (S7C Fig) and replication assays (S7D Fig). These results suggest that *Tg*6PGDH2 plays a vital role during the lytic cycle.

### *Tg*RPI is vital for *in vitro* and *in vivo* growth of tachyzoites

**Fig 4:**
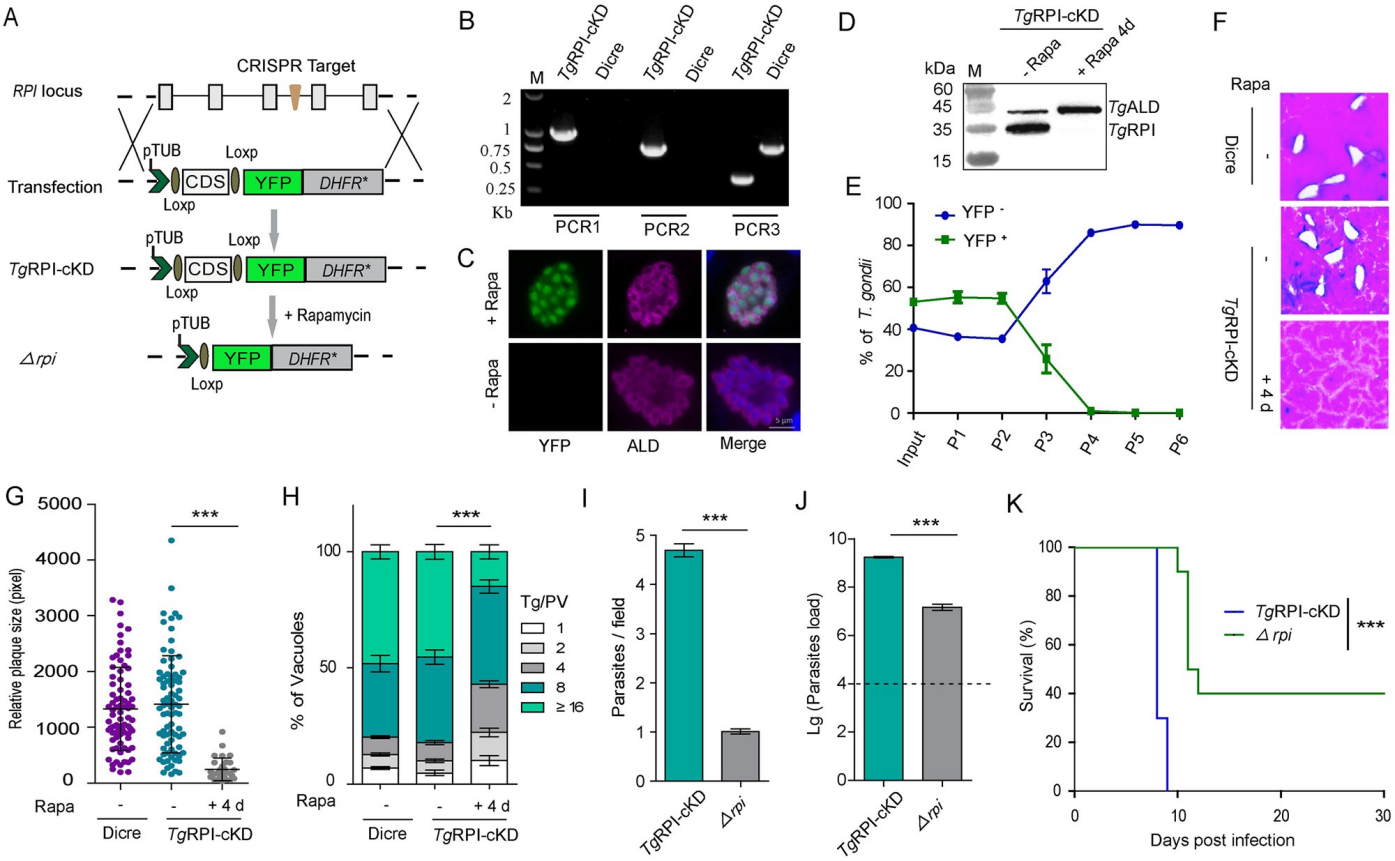
Depletion of *Tg*RPI results in severe growth defects *in vitro* and *in vivo*. **(A)**, Diagram showing strategy to construct the conditional knockout strain *Tg*RPI-cKD. **(B)**, Diagnostic PCRs on a representative *Tg*RPI-cKD clone. PCR1 and PCR2 checked the integration of homology templates at the 5^’^ and 3^’^ end of *TgRPI*, whereas PCR3 confirmed the deletion of endogenous *TgRPI* locus. **(C)**, Immunofluorescence staining for *Tg*ALD and YFP expression in *Tg*RPI-cKD parasites treated with rapamycin for 24 h. **(D)**, Western blotting for analyzing the expression of *Tg*RPI in *Tg*RPI-cKD parasites treated with or without rapamycin for 4 days. **(E)**, Competition assay comparing the growth of *Tg*RPI-cKD parasites treated with rapamycin for 24 h to that of untreated parasites. **(F-G)**, Deletion of *Tg*RPI showed severe lytic cycle defects by plaque assay and quantification of plaque sizes. Means ± SD of more than 60 plaques for each strain was graphed. **(H)**, Intracellular replication assay comparing parasite growth *in vitro*. *Tg*RPI-cKD parasites were treated with rapamycin for 4 days, and then they were allowed to infect fresh HFF cells for 24 h, subsequently, the number of parasites in each PV was checked by IFA. Results are means ± SEM for n = 3 independent experiments. **(I)**, Invasion assay where freshly egressed parasites were used to invade HFF monolayers for 20 min. Efficiencies of invasion, as determined by two-color staining to distinguish invaded vs non-invaded tachyzoites (means ± SEM, n = 3 assays), ***, *P<0*.*001*; Student’s t-test. **(J)**, Parasite loads in the peritoneal fluids of ICR mice. ICR mice were infected with *Tg*RPI-cKD and *Δrpi* tachyzoites (10^4^ tachyzoites/mouse) by intraperitoneal injection (n = 5 for each group), and parasite loads in peritoneal fluids 5 days post-infection were estimated by qPCR. **(K)**, Virulence tests of indicated strains in ICR mice (100 parasites per mouse, n = 10 mice for *Tg*RPI-cKD strain, n = 20 mice for *Δrpi* mutants), ***, *P<0*.*001*; Gehan–Breslow–Wilcoxon tests.

**Fig 5:**
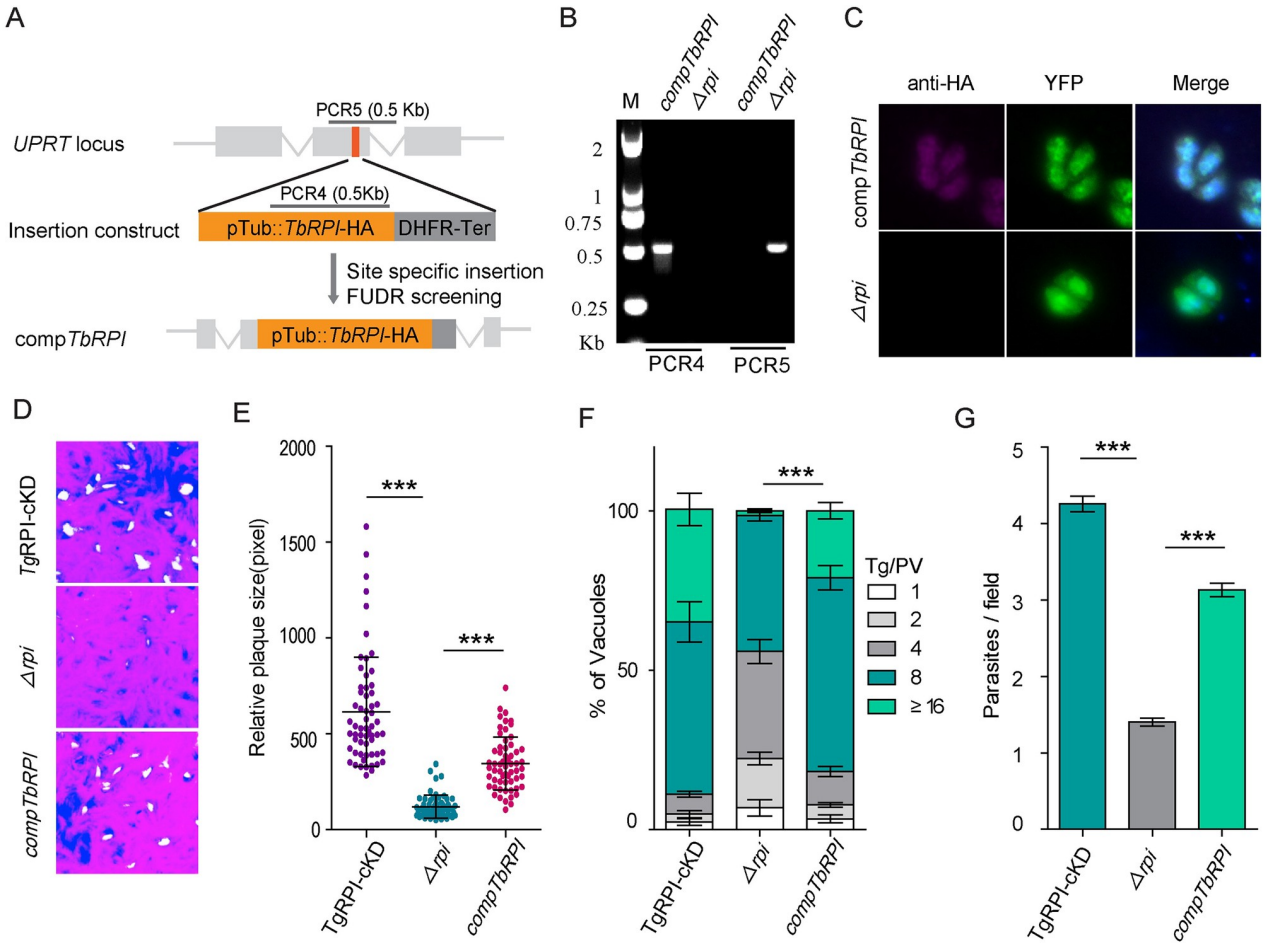
*Trypanosoma brucei* RPI complementation restored the growth defects of *Tg*RPI depletion mutants. **(A)**, Schematic diagram showing the insertion of a *Trypanosoma brucei* RPI expressing mini gene into the *UPRT* locus of the *Δrpi* strain by CRISPR/Cas9 mediated site-specific integration and selection with 10 μM FUDR. **(B)**, Diagnostic PCRs on a selected comp*Tb*RPI clone. PCR4 examined the successful insertion of *Trypanosoma brucei* RPI on the *UPRT* locus, whereas PCR5 confirmed the deletion of the endogenous *UPRT* locus. **(C)**, Expression of the complementing *Trypanosoma brucei* RPI as shown by IFA using rabbit anti-HA. **(D**-**E)**, Plaque assays comparing the growth of *Tg*RPI depletion strain before and after *Trypanosoma brucei* RPI complementation. Means ± SD of more than 60 plaques for each strain was graphed. Student’s t-test, ***, *P < 0*.*001*. **(F)**, Intracellular replication rates of depicted strains (24 h post-infection). Means ±SEM from three independent experiments (n = 3), each with two replicates. ***, *P < 0*.*001* by two-way ANOVA. **(G)**, The invasion efficiency of the comp*Tb*RPI was compared to the *Tg*RPI-cKD and *Δrpi* parasites. Means ±SD of more than 100 fields from three independent assays, ***, *P < 0*.*001*; one-way ANOVA.

We next investigated ribulose 5-phosphate isomerase (RPI), which serves the non-oxidative branch of the PPP. As described above, a conditional knockdown mutant of *Tg*RPI was generated and confirmed by PCR screening, immunofluorescence assay and Western blotting (Fig 4A–4D). The rapamycin-induced *Tg*RPI knockout parasites displayed an impaired phenotype *in vitro* as seen by competition (Fig 4E), plaque (Fig 4F and 4G) and replication assays (Fig 4H). To further determine the importance of *Tg*RPI, a *Tg*RPI-knockout (*Δrpi*) clonal mutant was produced from rapamycin-treated *Tg*RPI-cKD strain (S6C Fig). Western blotting confirmed the loss of *Tg*RPI expression in the *Δrpi* mutant (S6D Fig). The *Δrpi* parasites were viable and could be maintained *in vitro* but their ability to invade host-cell was significantly reduced (Fig 4I). The *Tg*RPI-cKD and *Δrpi* strains were used to infect ICR mice, and the parasite load in the peritoneal fluid was quantified by qPCR of the β-tubulin transcript (Fig 4J). The results suggested that *Tg*RPI plays a critical role in parasite propagation *in vivo*. To test the parasite virulence, we infected mice with *Tg*RPI-cKD and *Δrpi* tachyzoites, and monitored their survival for 30 days (Fig 4K). Consistent with *in vitro* data, knockout of *Tg*RPI resulted in attenuated virulence in mice. To decipher the catalytic function of *Tg*RPI, we complemented the *Δrpi* strain by expressing hemagglutinin (HA)-tagged *Trypanosoma brucei* RPI (*Tb*RPI) because the latter enzyme has been well-characterized [27]. The complemented strain comp*Tb*RPI was obtained after drug selection and clonal dilution (Fig 5A). The PCR and IFA (Fig 5B and 5C) confirmed the integration and expression of *Tb*RPI, respectively. Indeed, the growth and invasion defects were partly restored in the comp*Tb*RPI strain, as shown by plaque (Fig 5D and 5E), replication (Fig 5F) and invasion assays (Fig 5G). These data suggest that tachyzoites depend on RPI activity for the lytic cycle.

### *TgRPI* deletion perturbs the proteome of tachyzoites

**Fig 6:**
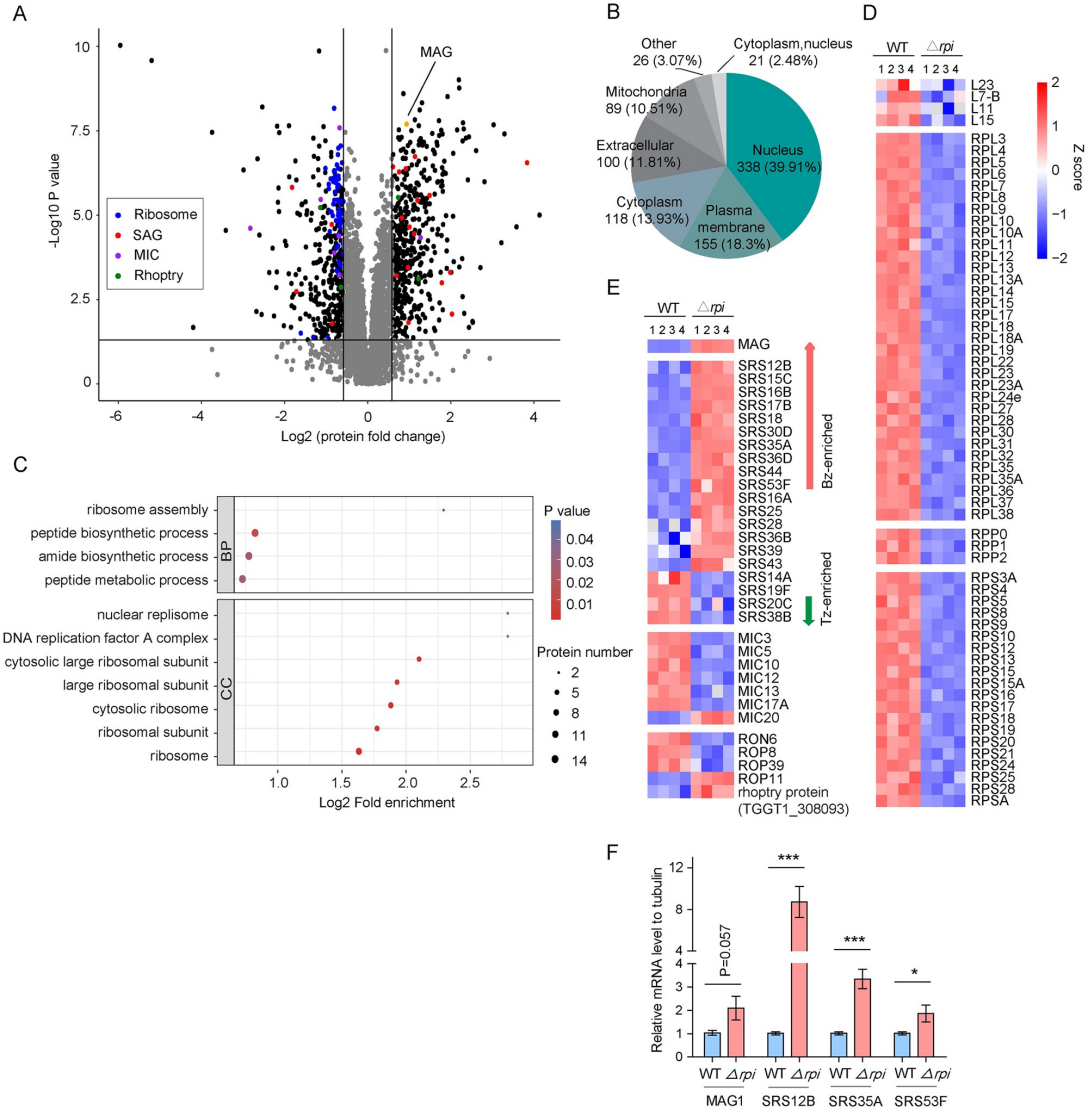
*Tg*RPI depletion alters the global proteomic profile. **(A)**, Volcano plots showing the protein expression difference based on 4D label-free quantitative proteomic data of *Δrpi* mutants. The X-axis shows log2 (1.5-fold change) versus the *Tg*RPI-cKD, and the Y-axis shows -log10 (*P* value) after ANOVA statistical test for n = 4 independent biological replicates. **(B)**, Putative subcellular localization of differentially expressed proteins. **(C)**, Enrichment and clustering analysis of the quantitative proteomics data sets based on gene ontology, only proteins that changed ≥1.5-fold in relative ratios (*P<0*.*05*) were considered. BP, biological process; CC, cellular component. **(D-E)**, Heat map of differentially expressed ribosomal proteins, microneme proteins, rhoptry proteins and stage-specific proteins. Bright red indicates a 1.5-fold change (*P<0*.*05*). White indicates no change. **(F)**, Four genes were selected for quantitative RT-PCR analysis, which examined their expression changes. The *β*-tubulin gene in parasites was used as an internal reference. Means ± SEM of three independent assays, *, *p <0* .*05*, ***, *P<0*.*001*; Student’s t-test.

To further enhance our understanding of the consequences of *Δrpi* and provide an integrative and global picture of the roles of ribose-5-phosphate in *Toxoplasma gondii* physiology, we also examined the effect of *Tg*RPI deletion on protein synthesis by 4D label-free mass spectrometry-based proteomics, which allowed identification and relative quantification of 3895 proteins. In total, 464 proteins were upregulated while 384 were downregulated in the *Δrpi* parasites. Significant repression of *Tg*RPI in the mutant validated the obtained data (Fig 6A). Subsequently, we analyzed the localization of proteins and found that many nuclear proteins were impacted upon depletion of *Tg*RPI (Fig 6B). The pathway enrichment analysis highlighted perturbation of proteins mainly involved in the peptide biosynthesis and ribosome assembly (Fig 6C). We observed a striking decrease in several ribosome constituent proteins including RPLs, RPPs and RPSs (Fig 6D), which are vital for protein synthesis. Other proteins affected in the *Δrpi* mutant included the micronemal and rhoptry proteins, some markedly decreased (Fig 6E), supporting our host-cell invasion data. Not least, we also noted a few bradyzoite-specific SRS increased, while tachyzoite-specific SRS were repressed. To validate these data, quantitative real-time PCR was used to analyze some differentially expressed genes between the wild type and *Δrpi* strain. As expected, the expression of *Tg*MAG1, *Tg*SRS12B, *Tg*SRS35A and *Tg*SRS53F was up-regulated in the *Δrpi* mutants (Fig 6F). Collectively, these results are indicative of slowed protein synthesis coupled with stage transition upon impairment of ribose 5-phosphate synthesis.

### *Tg*6PGDH2 and *Tg*RPI depletion impairs the flux of ^13^C-glucose in central carbon pathways

**Fig 7:**
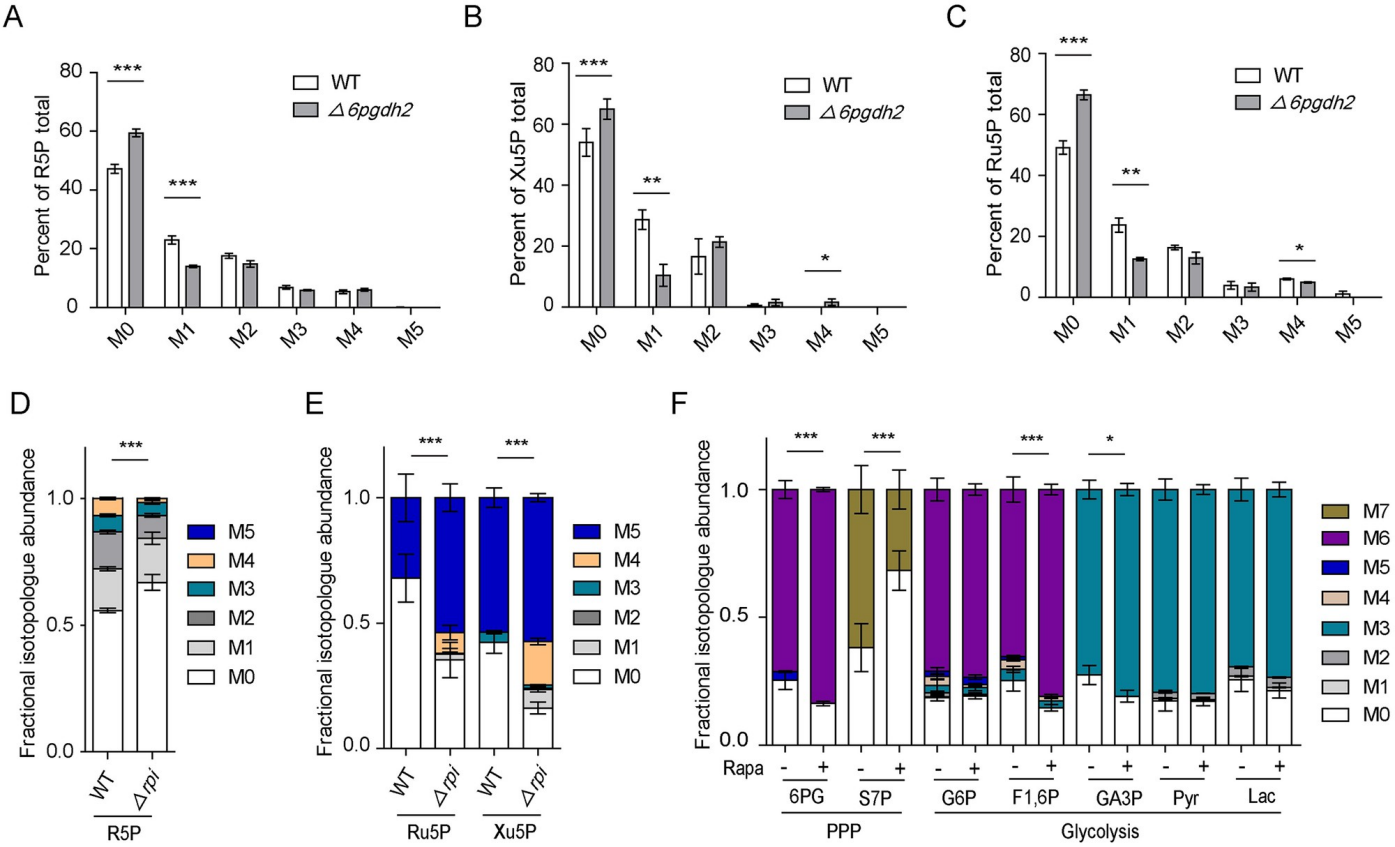
*Tg*6PGDH2 and *Tg*RPI are required to incorporate glucose-derived carbon into the pentose phosphate pathway. **(A-C)**, *Tg*6PGDH2-cKD (WT) and *Δ6pgdh2* mutants were propagated with HFF monolayers cultured in glucose-free DMEM medium supplemented with 8 mM 1,2-^13^C_2_-glucose for 12 h. Subsequently, intracellular parasites were collected, metabolites were extracted from the parasites, and the relative abundance of isotopologues was determined by LC-MS. M0 means parental unlabeled. M1-M5 represents the number of carbons in a selected metabolite labeled with ^13^C atom. Values are means ± SEM from four independent experiments (n = 4). Student’s t-test, *, *P<0*.*05*; **, *P<0*.*01*; ***, *P<0*.*001*. **(D)**, *Tg*RPI-cKD (WT), and *Δrpi* mutants were cultured the same way as above. Metabolite from the intracellular parasites was determined by LC-MS. Values are means ± SEM from five independent experiments (n = 5). ***, *P < 0*.*001* by two-way ANOVA. (**E**), Extracellular *Tg*RPI-cKD (WT) and *Δrpi* parasites were incubated in a glucose-free medium containing 8 mM [U-^13^C] glucose for 4 h. Incorporation of ^13^C into Ru5P and Xu5P was determined by UHPLC-HRMS platform. M0-M5 represents the number of carbons in a selected metabolite labeled with ^13^C atom. Values are means ± SEM from five independent experiments (n = 5). ***, *P < 0*.*001* by two-way ANOVA. **(F)**, Freshly egressed tachyzoites (3×10^7^) of *Tg*RPI-cKD left untreated or pretreated with rapamycin for 4 days were collected, syringe released, and then incubated in medium containing 8 mM [U-^13^C] glucose for 4 h. Incorporation of ^13^C into glycolysis and PPP intermediates was determined by UHPLC-HRMS platform. M0-M7 represents the number of carbons in a selected metabolite labeled with ^13^C atom. Values are means ± SEM from five independent experiments (n = 5). *, *P<0*.*05*; **, *P<0*.*01*; ***, *P <* .*001*; all by two-way ANOVA.

To interrogate whether *Tg6PGDH2* depletion affected pentose sugars synthesis, the intracellular *Tg*6PGDH2-cKD (WT) and *Δ6pgdh2* parasites were cultured with 1,2-^13^C_2_-glucose for 12 hours, followed by assessment by liquid chromatography-MS (LC-MS). The tracer labeling of fresh intracellular parasites showed that the M+1 labeled R5P in *Δ6pgdh2* mutants was significantly less than WT parasites (Fig 7A). We also observed that the M+1 labeled Xu5P and Ru5P containing one ^13^C were reduced in *Δ6pgdh2* mutants (Fig 7B and 7C). These data likely reflected an impaired pentose sugars synthesis upon *Tg*6PGDH2 depletion.

To investigate whether *TgRPI* deletion affected the metabolite abundances of PPP intermediates, the *Tg*RPI-cKD (WT) and *Δrpi* parasites were cultured in HFF cells and metabolite abundances were determined by LC-MS. Interestingly, although *TgRPI* deletion resulted in a marked reduction in growth rate, it did not significantly affect the metabolite abundances of several PPP intermediates (S8 Fig). To further reveal the metabolic consequences of genetic lesions, metabolic labeling of intracellular *Tg*RPI-cKD (WT) and *Δrpi* parasites was performed using 1,2-^13^C_2_-glucose. We found reduced incorporation of ^13^C into R5P upon *Tg*RPI depletion (Fig 7D). However, it should be noted that there was no significant change in the M+1 labeled R5P by *Tg*RPI disruption, which may be due to the metabolic complexity and the fact that R5P can also be produced by non-oxidized PPP. Because a previous study has demonstrated that the pentose phosphate cycle is non-negligible in the extracellular stage [4], we also performed [^13^C_6_]-glucose labeling in fresh extracellular parasites to analyze the incorporation of ^13^C into Ru5P and Xu5P. We found that the *Δrpi* parasites showed increased ^13^C labeling of Ru5P and Xu5P (Fig 7E). Similarly, the *Tg*RPI-cKD strain was cultured with or without rapamycin for 4 days, and fresh extracellular tachyzoites were labeled with [^13^C_6_]-glucose, followed by evaluation of selected metabolites using LC-MS. As expected, the inclusion of ^13^C into 6-phosphogluconate (6PG, an intermediate of oxidative branch) was significantly increased upon depletion of *Tg*RPI (Fig 7F). A reduction in the incorporation of ^13^C into sedoheptulose-7-phosphate (S7P, an intermediate of non-oxidative branch) was also observed (Fig 7F). In our extended work, we determined the glycolysis flux alterations upon depletion of *Tg*RPI. We found that the incorporation of ^13^C into fructose-1,6-bisphosphate and glyceraldehyde-3-phosphate was increased (Fig 7F). These data together indicated that a loss of *Tg*RPI impairs the operation of PPP and leads to an increase in glucose-fueled glycolysis.

## Discussion

**Fig 8:**
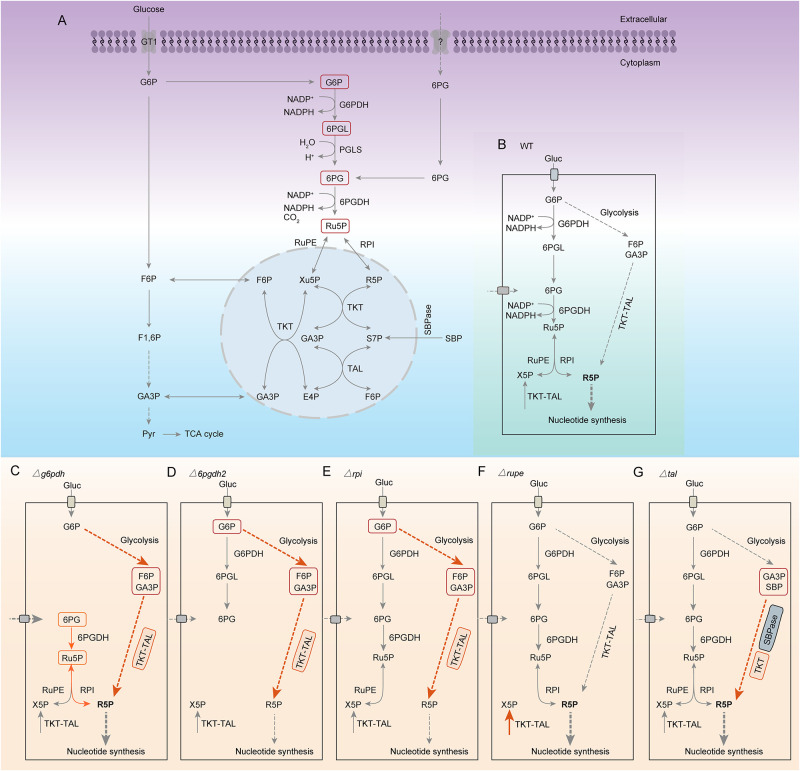
Proposed models for PPP metabolism under various conditions. **(A-B)**, Parasites can generate ribose-5-phosphate using the oxidative pentose phosphate pathway (*Tg*G6PDH-*Tg*6PGDH2-*Tg*RPI pathway) and non-oxidative pentose phosphate pathway (*Tg*TKT-*Tg*TAL pathway). **(C)**, Upon disruption of *Tg*G6PDH, *Tg*G6PDH-dependent pentose synthesis is blocked but ribose-5-phosphate is still produced by the intact downstream oxidative pentose phosphate pathway (*Tg*6PGDH2-*Tg*RPI pathway) which might utilize host-derived PPP intermediates. **(D)**, In *Δ6pgdh2* mutants, glucose imported from host cells cannot be fully catabolized to create ribose-5-phosphate. Even so, ribose-5-phosphate was still partly produced through the *Tg*TKT-*Tg*TAL pathway. **(E)**, Similar to *Δ6pgdh2* mutants, the *Δrpi* mutants also partly produced ribose-5-phosphate through the *Tg*TKT-*Tg*TAL pathway. **(F)**, Mutants lacking *Tg*RuPE rely on the *Tg*TKT-*Tg*TAL pathway for supplying Xylulose 5P. Notably, the ribose-5-phosphate can be generally provided by the oxidative and non-oxidative pentose phosphate pathway. **(G)**, In *Δtal* mutants, the ribose-5-phosphate can be provided through the oxidative pentose phosphate pathway and *Tg*TKT-*Tg*SBPase pathway.

This study shows that *Toxoplasma gondii* encodes a functional pentose phosphate pathway distributed in the cytoplasm and nucleus. *Tg*G6PDH1/2, *Tg*6PGDH1, *Tg*RuPE and *Tg*TAL are dispensable for parasite growth and virulence. Our work also reveals that *Tg*G6PDH-knockout tachyzoites usually grow but are susceptible to oxidative stress. By contrast, *Tg*6PGDH2 and *Tg*RPI are needed for the optimal growth of tachyzoites. In particular, depletion of *Tg*RPI impairs the metabolic flux of ^13^C-glucose and perturbs the parasite proteome. Our results suggest that PPP supports the core carbon metabolism by producing metabolic intermediates and NADPH in *T*. *gondii* (Fig 8A and 8B); However, the parasite can reprogram its carbon flux to maximize its survival upon genetic perturbation (Fig 8C–8G).

G6PDH as the first and rate-limiting enzyme of the PPP has an important function in various cells [28–31]. It has been reported previously that the knockdown of G6PDH inhibited the growth of cancer cells, such as leukemia THP-1 and human melanoma A375 cells [28,29]. Loss of G6PDH in *Trypanosoma brucei* indicated no obvious phenotype in the procyclic stage but was lethal for the bloodstream form [32]. Interestingly, G6PDH deletion in *Saccharomyces cerevisiae* appeared to grow normally [33]. We found that none of the two *Tg*G6PDHs is required for parasite development. Consistent with this finding, two *Tg*G6PDH genes have been assigned a positive phenotype score based on the genome-wide CRISPR-Cas9 screen (*Tg*G6PDH1 phenotype score = 0.69, *Tg*G6PDH2 phenotype score = 1.49) [34]. We speculate that ribose 5-phosphate in the *Δg6pdh1Δg6pdh2* mutant is provided through the transketolase-transaldolase (*Tg*TKT-*Tg*TAL) pathway, which can bridge the PPP and glycolysis by sharing GA3P and F6P (Fig 8C). Consistent with this, our attempts to delete *TgTKT* by CRISPR/Cas9-assisted homologous gene replacement were futile. On the other hand, disruption of *Tg*G6PDHs will block NADPH synthesis but maintain the intact downstream pathway. A previous study has reported that *Toxoplasma* expresses a phosphate transporter that plays a crucial role in the phosphate import [35]. There may be transporters in the parasite membrane to import 6-phosphogluconate and possibly other host-derived PPP intermediates that can sustain (low-level) synthesis of NADPH in the *Δg6pdh1Δg6pdh2* strain. In addition, there remains a possibility of the remaining-G6PDH activity is not encoded by *Tg*G6PDH1 and *Tg*G6PDH2. Previous studies determined that tracing deuterium isotope from [3-^2^H] glucose could reveal the contribution of the oxidative PPP enzymes to the cellular NADPH pool [36]. Therefore, using [3-^2^H] glucose to label NADPH would be an excellent way to assess NADPH production by *Tg*G6PDH or other sources. It is plausible that other NADPH-generating enzymes (such as isocitrate dehydrogenase, malic enzyme and transhydrogenase) may contribute synergistically in *T*. *gondii* [37,38]. NADPH is required to protect mammalian cells against the oxidative stress [24–26]. Likewise, we observed that the replication of the H_2_O_2_-treated double mutant was notably impaired, which indicates a physiological role of G6PDH in NADPH homeostasis and under oxidative stress.

Our bioinformatic search did not find annotation for the second enzyme of the PPP (6-phosphogluconolactonase or 6PGL) in *T*. *gondii*. The related apicomplexan parasite *Plasmodium falciparum* harbors a bifunctional G6PDH-6PGL catalyzing the first two reactions [39]. It is, therefore, possible that *Tg*G6PDH1/2 encodes 6PGL activity that requires further investigation. This study also reports that two *Tg*6PGDH enzymes in *T*. *gondii*, one residing in the cytoplasm (*Tg*6PGDH2) play a crucial role in the parasite growth. In contrast, the other protein (*Tg*6PGDH1) is not expressed during the lytic cycle. Our data indicated that tachyzoites require *Tg*6PGDH2 to produce ribulose-5-phosphate (Ru5P), which in turn yields ribose 5-phosphate (R5P). Upon deletion of *Tg*6PGDH2, the *Tg*TKT-*Tg*TAL pathway may become the primary pathway for generating R5P despite low-efficiency (Fig 8D). *Tg*6PGDH1 is dispensable *in vitro* and *in vivo*, which is anticipated because this isoform is not expressed in tachyzoites. Interestingly, *Tg*6PGDH1 appears up-regulated in sporulated oocysts, implying its vital role in the sporozoite formation [40].

In the non-oxidative branch of the PPP, RPI supports ribose-5-phosphate synthesis. Its inactivation severely inhibits the tachyzoite growth, suggesting that the *Tg*TKT-*Tg*TAL pathway cannot adequately compensate for its loss in the *Tg*RPI-cKD mutant (Fig 8E). *Tg*RuPE was dispensable for yielding X5P, which consistent with the CRISPR/Cas9 screen (phenotype score = -0.31) [34]. We reasoned that the *Δrupe* tachyzoites might supply X5P by the *Tg*TKT-*Tg*TAL pathway (Fig 8F). Moreover, *Tg*TAL was not needed for the parasite growth and virulence, as the *Δtal* mutant may obtain S7P by *Tg*SBPase, which removes one phosphate group from sedoheptulose-1,7- bisphosphate (SBP) to produce S7P (Fig 8G). Previous studies showed that deletion of *Tg*SBPase reduced the fitness of *T*. *gondii* [17].

Ribose 5-phosphate (R5P) is a vital source for de novo synthesis of nucleotide and amino acid [41]. In this study, we reasoned that *Tg*RPI depletion impairs carbon metabolic homeostasis and hinders R5P production, which may result in transcribing and translating tardily, affecting regular protein biosynthesis. This hypothesis was further supported by our quantitative proteomic data which displayed that the biosynthesis of ribosomal proteins, micronemal proteins, rhoptry proteins and other proteins were significantly affected (Fig 6). Our quantitative proteomic analysis also showed that long-term *Tg*RPI-depleted caused a few bradyzoite-specific SRS increases while tachyzoite-specific SRS were repressed, which perhaps hinting that impairing ribose 5-phosphate synthesis caused slow growth and triggered stage conversion. Consistent with quantitative proteomic data, the up-regulated expression of *Tg*MAG1, *Tg*SRS12B, *Tg*SRS35A and *Tg*SRS53F in the *Δrpi* mutants was further validated by quantitative real-time PCR. Type I *T*. *gondii* strain (RH) has less tendency for conversion to bradyzoites. Because *Δrpi* mutants were generated in the RH strain, it would be very insightful to analyze the bradyzoite conversion of *TgRPI* deletion mutants in more cystogenic type II parasites (ME49) in further study.

Based on bioinformatics analysis, combined with phenotypic features of *Tg*6PGDH2 and *Tg*RPI knockout strains *in vitro* and *in vivo*, we speculate that *Tg*6PGDH2 and *Tg*RPI are potential targets for drug designs against *Toxoplasma* infections. Our bioinformatics analysis found that although the amino acid sequence of *Tg*6PGDH2 and homo sapiens 6PGDH (*Hs*6PGDH) share about 51% identity, its C-terminal sequences diverge from that of *Hs*6PGDH. The amino acid sequence of *Tg*RPI was more similar to that of *Plasmodium falciparum* RPI, with only 42% identity with homo sapiens RPI. Especially, encouraged by selective inhibition of *T*. *brucei* 6PGDH by hydroxamic derivatives [42], we thought it would be interesting to screen inhibitors of *Tg*6PGDH2 in future studies. In addition, the crystal structure of *Tg*RPI has been identified [22], which provided reference information for the design of inhibitors against *Tg*RPI.

In conclusion, our findings delineate the pentose phosphate pathway in *T*. *gondii*, and establish its physiological importance in the parasite metabolism, reproduction and virulence. Moreover, *Tg*6PGDH2 and *Tg*RPI emerge as the two potential therapeutic targets against toxoplasmosis. Not least, we further highlight the metabolic plasticity in tachyzoites that enables them to survive.

## Materials and methods

### Ethics statement

All animal experiments were approved by the Ethical Committee of South China Agricultural University (permit no.2021f146).

### Mice and parasite strains

Seven-week-old female ICR mice were purchased from the Guangdong Medical Experimental Animal Center in Guangdong Province. They were maintained under standard conditions according to the regulations of the Administration of Affairs Concerning Experimental Animals.

The RH*Δku80* and DiCre strains of *T*. *gondii* used in this study were grown within human foreskin fibroblast (HFF) cells (ATCC, Manassas, VA, USA). Cultures were done in Dulbecco’s modified Eagle’s medium (DMEM) supplemented with 10% fetal bovine serum (Gibco Life Technologies, Inc., Rockville, MD, USA), 10 U/ml penicillin and 100 μg/ml streptomycin. All genetically modified strains were generated from the parental strains and maintained *in vitro* under the same growth conditions as parental strains.

### Plasmid construction

All primers used for each reaction and plasmids used in the mutant constructions are listed in S1 and S2 Tables, respectively. Locus-specific CRISPR/Cas9 plasmids were generated using the Q5 site-directed mutagenesis kit (New England Biolabs Inc., USA) as described previously [43]. Other plasmids were constructed by multifragment ligation using the ClonExpress II one-step cloning kit (Vazyme Biotech Co., Ltd, Nanjing, China). All plasmids were verified by DNA sequencing before use.

### Parasite culturing and transfection

The corresponding locus-specific CRISPR/Cas9 plasmids and homologous donor templates (S2 Table) were co-transfected into freshly tachyzoites of the RH*Δku80*, Dicre [44,45] or derivative strains, selected with 1 μM pyrimethamine (Sigma-Aldrich, USA) or 30 μM chloramphenicol (Sigma-Aldrich, USA), and single cloned by limiting dilution. The *Tb*RPI complement strain was constructed by cotransfecting the *UPRT*-specific CRISPR plasmids along with the *Tb*RPI-expressing cassettes into the *Δrpi* mutants and selected with 10 μM 5-flurodeoxyuracil (FUDR). The comp*Tg6PGDH2* strain was constructed by inserting a *Tg*6PGDH2 expressing cassette into the *UPRT* locus of the *Tg*6PGDH2-cKD strain and selected with FUDR. Positive clones were identified by diagnostic PCRs (primers listed in S1 Table). All transgenic parasites used in this study are listed in S3 Table.

### Polyclonal antibodies production and immunofluorescence assays

The *Tg6PGDH2* and *TgRPI* were amplified from the cDNA of the RH*Δku80* strain and cloned into the vector pCold. Subsequently, recombinant proteins *Tg*RPI and *Tg*6PGDH2 were purified from *E*. *coli* BL21 (DE3). The expression of proteins was tested by SDS-PAGE and western blot analyses. The purified recombinant proteins *Tg*G6PDH2 and *Tg*RPI were used to immunize 7-week-old female mice respectively. Positive antiserum was collected and stored at -80°C. To check the localization of native *Tg*G6PDH2 and *Tg*RPI, the intracellular RH*Δku80* tachyzoites were purified and used to infect HFF cells. Then, the cells were fixed, permeabilized, and blocked. Next, the coverslips were incubated with rabbit anti-*Tg*ALD (a cytoplasm marker) and mouse anti-*Tg*G6PDH2 or mouse anti-*Tg*RPI, stained with Alexa Fluor 488 goat anti-mouse IgG, Alexa Fluor 594 goat anti-rabbit IgG secondary antibodies and Hoechst and then imaged under fluorescence microscopy on a BX53 microscope (Olympus, Tokyo, Japan).

### Western blot assay

The *Tg*6PGDH2-cKD and *Tg*RPI-cKD parasites were grown in a DMEM medium with or without rapamycin treatment for 4 days. The parasites of *Δrpi* and *Δ6pgdh2* were cultured under standard tissue culture conditions. Then all parasites were purified and collected respectively, and the whole proteins of each mutant were extracted with lysis buffer. Finally, the corresponding protein expression was detected by Western blot using mouse anti-*Tg*6PGDH2 or mouse anti-*Tg*RPI. The rabbit anti-*Tg*ALD was used as an internal reference. Finally, the signals were visualized with the super ECL detection reagent on UVP ChemStudio (Analytik Jena, Germany).

### Plaque assay

HFF monolayers seeded on 6-well plates were infected with freshly egressed parasites (100 tachyzoites/well, three wells per strain). Subsequently, the plates were cultured at 37°C with 5% CO_2_ for 7 or 9 days. Then they were fixed with 4% paraformaldehyde, stained with 0.1% crystal violet for 20 minutes and imaged on a scanner (Microtek Scan Marker i600, MICROTEK, China) to analyze the relative sizes and number of plaques, as described previously [6].

### Intracellular replication assay

Parasites were used to infect fresh HFF cells seeded on coverslips (two wells per strain) for 1 h. Subsequently, the extracellular parasites were washed away using PBS, and the rest of the cultures were kept at 37°C with 5% CO_2_ for another 24 h. Cells were fixed with 4% paraformaldehyde, incubated with rabbit anti-*Tg*ALD for 20 min, permeabilized with 0.1% Triton X-100 for 15 min, incubated with mouse anti-*Tg* IgG for 20 min and stained with Hoechst, Alexa Fluor 488-conjugated goat anti-mouse lgG secondary antibodies and Alexa Fluor 594-conjugated goat anti-rabbit lgG secondary antibodies for 20 min. Following fluorescence staining, a minimum of 150 vacuoles were examined for each sample by fluorescence microscopy to determine the number of parasites in each parasitophorous vacuole.

### Analysis of H_2_O_2_ resistance

Freshly egressed RH*Δku80*, *Δg6pdh1*, *Δg6pdh2* and *Δg6pdh1Δg6pdh2* tachyzoites were incubated for 3 h in DMEM containing 500 μM H_2_O_2_ as described previously [46,47]. Then parasites were used to infect fresh HFF cells for 2 h, and invaded parasites were grown under standard growth conditions for another 24 h. Subsequently, the samples were fixed, stained and analyzed according to the protocols specified in the above intracellular replication assay.

### NADPH assay

All parasites (approximately 3x10^7^ cells/strain) were first purified and collected. Strains were then washed with cold PBS and collected by rotating at low speed for 5 minutes. Subsequently, the NADPH was detected using the NADP/NADPH assay kit (Abcam, Cambridge, UK). Briefly, 400 μl NADP/NADPH lysis buffer was added to extract parasites by carrying out two freeze cycles (20 min on dry ice followed by 10 min at room temperature). Then the extractions were centrifuged, and 200 μl supernatants of each sample were heated at 60°C for 30 min and cooled down on ice immediately to decompose the NADP^+^. Added 100μl of the reaction mix to each standard, sample well, and incubated the plate at room temperature for 5min. At last, 10 μl of NADPH developer was added to each well and mixed. After 4 hours, the optical density of samples was measured at OD450 nm by a SYNERGY multi-mode reader (BioTek Instruments, Inc, USA).

### Competition assay

The *Tg*6PGDH2-cKD and *Tg*RPI-cKD parasites were pretreated with or without 50 nM rapamycin for 24 h. Then pretreated and untreated parasites were harvested, mixed in approximately 1:1 ratio, and YFP positive parasites were monitored every 2 days by flow cytometry. About 10, 000 events per sample were acquired on a CytoFLEX (Beckman Coulter, Inc., USA) and data analysis was proceeded using CytExpert software.

### Invasion assay

Freshly egressed parasites were purified, counted and then used to infect HFF cells (10^6^ tachyzoites/strain) for 20 min at 37°C with 5% CO_2_. Subsequently, the samples were washed with PBS, fixed with 4% paraformaldehyde, incubated with rabbit anti-*Tg*ALD for 20 min, permeabilized with 0.1% Triton X-100 for 15 min and blocked with 10% FBS. The *Tg*RPI-cKD strain was incubated with mouse anti-*Tg* IgG for 20 min while *Δrpi* and *compTb*RPI strains with YFP-positive omitted this step. Subsequently, the *Tg*RPI-cKD strain was stained with Hoechst, Alexa Fluor 488 goat anti-mouse lgG secondary antibodies and Alexa Fluor 594 goat anti-rabbit lgG secondary antibodies, while *Δrpi* and comp*TbRPI* strains were stained with Hoechst and Alexa Fluor, 594-conjugated goat anti-rabbit lgG secondary antibodies for 20 min. After fluorescence staining, the number of parasites invading cells in each field was counted by fluorescence microscope. At least 150 fields were observed in each sample.

### Virulence testing

Seven-week-old female ICR mice were injected intraperitoneally with 100 freshly egressed tachyzoites. The symptoms and survival of mice were monitored for 30 days, and blood samples from mice that survived were collected afterwards. Mice seronegative by IFA or enzyme-linked immunosorbent assay were not included in the analysis [19]. Cumulative mortality was plotted as Kaplan-Meier survival plots and analyzed using Prism 5 (GraphPad Software Inc., La Jolla, CA, USA).

### Determination of parasite burden in peritoneal fluids of mice

Freshly egressed 10^4^ tachyzoites were allowed to infect female ICR mice (7 weeks old) by intraperitoneal injection. Five days post-infection, the mice were euthanized, their peritoneal fluids were collected and genomic DNA was extracted using the TIANamp Blood DNA Kit (Tiangen Biotech Co. Ltd, Beijing, China). Parasite burden in peritoneal fluids was determined by quantitative PCR (primers listed in S1 Table), as described previously [19].

### Whole genome sequencing

Freshly egressed *Δg6pdh1Δg6pdh2* tachyzoites were purified and genomic DNA from the parasites was extracted using the TIANamp Blood DNA Kit (Tiangen Biotech Co. Ltd, Beijing, China). Subsequently, purified genomic DNA was subject to genome sequencing as described previously [48]. Clean reads were mapped to the reference genome of the *T*. *gondii* GT1 strain. The mapping results were visualized by the Integrative Genomics Viewer (https://software.broadinstitute.org/software/igv/).

### Semi-quantitative RT-PCR and quantitative real-time PCR

All freshly egressed tachyzoites (RH*Δku80*; *Δg6pdh1*; *Δg6pdh2*; *Δg6pdh1Δg6pdh2*; *Δrupe*; *Δtal*; *Tg*RPI-cKD; *Δrpi*) were purified using 3 μm polycarbonate membranes and collected firstly. Then total RNA was extracted from each mutant by Eastep super total RNA extraction kit (Promega Biotech Co. Ltd, Beijing, China) and reversely transcribed to cDNA referring to the method of cDNA synthesis kit (Yeasen Biotechnology Co., Ltd., Shanghai, China). To validate the successful gene knock-out of each mutant, semi-quantitative PCR was performed using equivalent cDNA. Besides, Transcript levels for *Tg*G6PDH1, *Tg*G6PDH2, *Tg*MAG1, *Tg*SRS12B, *Tg*SRS35A and *Tg*SRS53F in each sample were analyzed by quantitative real-time PCR with SYBR Green PCR mix (TOYOBO, Osaka, Japan) in a Light Cycler 480 (Roche, Basel, Switzerland), using β-tubulin as an internal reference. Primers used for semi-quantitative RT-PCR and real-time PCR are listed in S1 Table in the supplemental material.

### Transcriptome analysis

The tachyzoites of RH*Δku80* and *Δg6pdh1Δg6pdh2* were collected and purified. Then total RNA from each sample was extracted using Transzol UP Reagent (TransGen Biotech Co., Ltd, Beijing, China) according to the manufacturer’s instructions. Subsequently, RNA sequencing was performed as described previously [49]. Briefly, mRNA was purified and captured by magnetic beads with Oligo (OT) and then was fragmented. Random primers were used for reverse transcription to synthesize the first strand of cDNA, and second-strand synthesis was combined with A-tailing. The quality of libraries was assessed by qPCR, and the qualified libraries were sequenced by the Illumina platform with the PE150 strategy. Then clean reads were obtained by removing low-quality sequences and were analyzed using three analytical processes: sequencing data quality control, data comparison analysis, and transcriptome deep analysis. In the end, classification and feature analysis were performed according to different genomic annotation information. Then the expression levels of each mutant were calculated, and differential expression analysis was performed. The transcriptome data analysis was performed using the online platform of Majorbio Cloud Platform (www.majorbio.com).

### Proteomic analyses

All parasites (3x10^7^ parasites/strain) were sonicated using an ultrasonic processor in lysis buffer (1% SDS,1% protease inhibitor cocktail) and centrifuged at 4°C at 12000 g for 10 min. Then the supernatant was collected and the protein concentration was determined with a BCA kit (Beyotime Biotechnology, Shanghai, China) according to the manufacturer’s instructions. Subsequently, the supernatants were precipitated with cold acetone at -20°C for 2 h. After centrifuging at 4500 g at 4°C for 5 min, the remaining precipitates were washed with cold acetone (Zhejiang Hannuo Chemical Technology Co., Ltd., Lanxi City, Zhejiang Province, China) twice and redissolved in 200 mM tetraethylammonium bromide (Sigma-Aldrich, St. Louis, Missouri, USA). Then the protein was hydrolyzed overnight with trypsin (at a 1:50 mass ratio of trypsin to protein). The next day, the protein solution was reduced with 5 mM DL-dithiothreitol (Sigma-Aldrich) for 30 min at 56°C and alkylated with 11 mM iodoacetamide (Sigma-Aldrich) at room temperature in the dark for 15 min. The peptides obtained by enzymolysis were first dissolved in solvent A (aqueous solution of 0.1% formic acid and 2% acetonitrile) and then were transferred into a home-made reversed-phase analytical column with a length of 25 cm and an inner diameter of 75/100 μm. At last, the peptides were separated with solvent B (aqueous solution containing 0.1% formic acid and 99.9% acetonitrile) by using UltiMate 3000 UHPLC system (liquid gradient setting: from 6% to 24% solvent B in 70 min, from 24% to 35% in 14 min, and went up to 80% in 3 min then stayed at 80% for 3min; flow rate: constant current, maintained at 450 nL/min). The peptides were subjected to a capillary source followed by the timsTOF Pro mass spectrometry (Bruker Daltonics Inc., USA) with the mode of parallel accumulation serial fragmentation (PASEF). Fragments and precursors were analyzed utilizing a TOF detector and the MS/MS scan ranged from 100 to 1700 m/z. Precursors were selected for fragmentation with charge states 0 to 5, and PASEF-MS/MS scans were performed 10 times per cycle. Besides, set the dynamic exclusion time to 30s. Subsequently, a sample-specific protein database was constructed based on the samples, and the quality control analysis was performed on the peptide and protein levels. Then, the GO, KEGG and other databases were used to annotate standard functions of identified proteins and quantitative analysis of proteins was performed. According to the quantitative results, differential screening and functional classification statistical analysis of differential proteins were conducted. Finally, fisher’s exact test was used to analyze the statistical results of rich cluster analysis to compare the functional relationship of differential proteins under different experimental conditions.

### Metabolic analysis

The *Tg*RPI-cKD parasites were grown in the corresponding medium with or without rapamycin treatment for 4 days. The tachyzoites of RH*Δku80*, *Δg6pdh1Δg6pdh2* and *Δrpi* were grown *in vitro* under standard tissue culture conditions. The freshly egressed parasites were filtered using 3 μm polycarbonate membranes. Then 3x10^7^ parasites were cultured in a glucose-free DMEM medium supplemented with 8 mM ^13^C_6_-glucose for 4 h. After that, the parasites were washed with PBS and lysed in 1 ml of ice-cold methyl alcohol, as described previously [6,19]. Then the samples were treated with 5 cycles of "1 min ultrasound and 1 min interval" on an ice bath and placed at -20°C for 30 min. After that, the supernatant centrifuged was evaporated with nitrogen and redissolved in 50% aqueous acetonitrile. Then, chromatographic separation was performed on the Ultimate 3000 UHPLC system (Thermo Fisher Scientific, USA) with a Waters BEH Amide column (2.1 mm × 150 mm, 1.7 μm). The mobile phase consisted of (A) water containing 15 mM ammonium acetate (pH = 8.5) and (B) acetonitrile/water (90:10, volume ratio) and the flow rate was set at 0.35 mL/min. Subsequently, the metabolites were eluted in a linear gradient mode with the following program: 0–2 min, 90% B; 14 min, 75% B; 15 min, 65% B; 15.2–16.9 min, 50% B; 17–20 min, 90% B. At last, the eluents were analyzed in heated electrospray ionization negative (HESI-) mode using Q Exactive Hybrid Quadrupole-Orbitrap Mass Spectrometry (Thermo Fisher Scientific, USA) and the parameters were set as follows: Spray voltage: 3500 V; Scan rage: mass/charge ratio: 70–1050 and AGC:1× 10^6^. At last, the data was analyzed with Xcalibur software, and the raw mass spectrometry data was corrected using IsoCor v2 as described previously [50].

Quantification of PPP metabolites by UHPLC-MS. The *Tg*RPI-cKD (WT) and *Δrpi* parasites were processed as described above, except for the evaporated residues redissolved in 40 μL of 50% aqueous acetonitrile with 5 μg/mL of ^13^C_6_-F1,6P to UHPLC-HRMS analysis. The raw mass spectral data were acquired using Xcalibur software, and relative abundances of PPP intermediates were obtained using the added ^13^C_6_-F1,6P as a reference.

Labeling of PPP intermediates from 1,2-^13^C_2_-glucose in intracellular parasites. The intracellular *Tg*6PGPDH2-cKD, *Δ6pgdh2*, *Tg*RPI-cKD and *Δrpi* were grown in a glucose-free DMEM medium containing 8 mM 1,2- ^13^C_2_-glucose (Sigma-Aldrich, USA) for 12 h and approximately 3x10^7^ parasites were collected. Then the samples of each strain were prepared as described previously [6,19,51] and redissolved in 50 μL of 10% methanol with 0.1% formic acid. Subsequently, the LC-MS/MS analysis was performed on an Agilent 1290 Infinity II UHPLC system coupled to a 6470A triple quadrupole mass spectrometry (Santa Clara, CA, United States) as described previously [51]. Chromatography was performed using a Waters HSS T3 column (2.1 mm × 100 mm, 1.7 μm). The instrument setting parameters of LC-MS/MS was almost the same as those above, except that the flow rate was changed to 0.25 mL/min, and the chromatographic separation gradient elution procedure was changed to 0–1 min, 1% B; 2 min, 10% B; 7 min, 10% B; 9 min, 99% B. 11 min, 99% B; 11.1 min, 1% B and kept until 13 min. While the main parameters of the ion source were changed as follows: the spray voltage was 3000 V. The capillary temperature and the Probe heater temperature were 300°C and 350°C respectively. The sheath gas flow rate was changed to 11 L/min. Finally, data were acquired using the MassHunter software (version B.08.00, Agilent), and the raw MS data was corrected by IsoCor v2 as described previously [50].

### Statistical analysis

All statistical analyses were performed in GraphPad Prism 5 (GraphPad Software Inc., La Jolla, CA, USA) using Student’s t-tests, Gehan–Breslow–Wilcoxon tests, one-way analysis of variance (ANOVA) with Bonferroni post-tests, or two-way ANOVA as indicated in the figure legends.

## Supporting information

S1 Fig Expression and localization of *Tg*6PGDH2. A-B, SDS-PAGE and western blot analysis of his-*Tg*6PGDH2 recombinant protein. M: molecular weight markers; lane 1: lysate from recombinant bacteria without IPTG induction; lane 2: lysate from IPTG-induced recombinant bacteria; lane 3: Supernatant of his-*Tg*6PGDH2 protein; lane 4: Inclusion body of his-*Tg*6PGDH2 protein. C, Immunofluorescent microscopic analysis of co-localization of native *Tg*6PGDH2 with the cytoplasmic marker, *Tg*ALD.
(TIF)

S2 Fig Expression and localization of *Tg*RPI. A-B, SDS-PAGE and western blot analysis of his-*Tg*RPI recombinant protein. M: molecular weight markers; lane 1: lysate from recombinant bacteria without IPTG induction; lane 2: lysate from IPTG-induced recombinant bacteria; lane 3: Supernatant of his-*Tg*RPI protein; lane 4: Inclusion body of his-*Tg*RPI protein. C, Immunofluorescent microscopic analysis of co-localization of native *Tg*RPI with the cytoplasmic marker, *Tg*ALD.
(TIF)

S3 Fig Construction of *Tg*G6PDH1, *Tg*G6PDH2, *Tg*6PGDH1, *Tg*RuPE and *Tg*TAL deletion strains. **(A)**, Scheme showing the generation of the *Δg6pdh1*, *Δg6pdh2*, *Δ6pgdh1*, *Δrupe* and *Δtal* mutants via CRISPR/Cas9-assisted gene editing. **(B-F)**, Diagnostic PCRs confirming the *Δg6pdh1*, *Δg6pdh2*, *Δ6pgdh1*, *Δrupe* and *Δtal* mutants. **(G-J)**, Semi-quantitative RT-PCR confirming the *Δg6pdh1*, *Δg6pdh2*, *Δrupe* and *Δtal* mutants. The *β*-tubulin was included as a control.
(TIF)

S4 Fig Generation of a *Tg*G6PDH1 and *Tg*G6PDH2 double-deletion strain. **(A)**, Schematic illustration of knocking out *Tg*G6PDH1 in the *Δg6pdh2* strain to produce *Δg6pdh1Δg6pdh2* mutant. **(B)**, Diagnostic polymerase chain reaction (PCRs) for a *Δg6pdh1Δg6pdh2* mutant clone. **(C)**, Semi-quantitative RT-PCR confirming the *Δg6pdh1Δg6pdh2* mutant. The *β*-tubulin was included as a control. **(D-E)**, Confirmation of *Tg*G6PDH1 and *Tg*G6PDH2 double-deletion by whole genome sequencing. Genomic DNA was extracted from *Δg6pdh1Δg6pdh2* mutants and subject to genome sequencing. Subsequently, the clean reads were mapped to the reference genome of the GT1 strain and visualized by the Integrative Genomics Viewer. **(F)**, Volcano plot comparing the log2 (fold change) gene expression for the *Δg6pdh1Δg6pdh2* mutants versus the RH*Δku80* strain under standard growth conditions. Significantly downregulated genes were shown in blue (*P<0*.*05*).
(TIF)

S5 Fig Incorporation of [U-^13^C] glucose-derived carbon into PPP, glycolysis and TCA cycle intermediates. Extracellular tachyzoites of the *Δg6pdh1Δg6pdh2* and parental strain were collected, purified, and then incubated in a medium containing 8 mM [U-^13^C] glucose for 4 h, followed by metabolite extraction and LC-MS analysis. WT (RH*Δku80*), *Δ* (*Δg6pdh1Δg6pdh2*). Values are means ± SEM from five independent experiments (n = 5). *, *P<0*.*05*; **, *P<0*.*01*; ***, *P <* .*001*; all by two-way ANOVA.
(TIF)

S6 Fig Generation of *Tg6PGDH2* and *TgRPI* deletion mutants. **(A)**, *Tg*6PGDH2-cKD strain was treated using rapamycin for 24 h and then one clean *Tg6PGDH2*-knockout clone (*Δ6pgdh2*) was produced through limiting dilution. Diagnostic PCR on a selected *Δ6pgdh2* clone confirming the deletion of *Tg6PGDH2*. **(B)**, Loss of *Tg*6PGDH2 expression in the *Δ6pgdh2* mutant as checked by Western blotting using mouse anti-*Tg*6PGDH2 and rabbit anti-*Tg*ALD. *Tg*ALD was included as a loading control. **(C)**, Diagnostic PCR on a selected *Δrpi* clone confirming the deletion of *TgRPI*. Primers used for diagnostic PCR validation were listed in S1 Table. **(D)**, Western blotting using mouse anti-*Tg*RPI and rabbit anti-*Tg*ALD for checking the expression of *Tg*RPI.
(TIF)

S7 Fig Construction and characterization of a *Tg*6PGDH2 complementing strain. **(A)**, Schematic illustration showing the insertion of a *Tg*6PGDH2 expressing cassette into the *UPRT* locus of the *Tg*6PGDH2-cKD strain by CRISPR/Cas9-assisted site-specific integration and selection with 5-fluorodeoxyuridine (FUDR). **(B)**, Diagnostic PCRs on a selected comp*Tg6PGDH2* clone. PCR1 and PCR2 examined the integration of homology templates at the 5^’^ and 3^’^ end of *Tg6PGDH2*, whereas PCR3 confirmed the deletion of the endogenous *UPRT* locus. **(C)**, Plaque assays comparing the growth of *Tg*6PGDH2-cKD strain and comp*Tg6PGDH2* strain. **(D)**, Intracellular replication rates of depicted strains (24 h post-infection). *Tg*6PGDH2-cKD strain and comp*Tg6PGDH2* parasites were pretreated with or without rapamycin for 6 days, and then intracellular replication of parasites was measured, as determined by the number of parasites in each parasitophorous vacuole (Tg/PV). Means ±SEM from three independent experiments (n = 3). NS = not significant, ***, *P < 0*.*001* by two-way ANOVA.
(TIF)

S8 Fig Relative abundances of selected metabolites. The intracellular *Tg*RPI-cKD (WT) and *Δrpi* parasites were collected and purified using a 3 μm pore size filter. Then 3x10^7^ parasites were lysed with ice-cold methyl alcohol and metabolite abundances were measured by LC-MS. Relative concentrations of indicated metabolites were calculated using the added ^13^C_6_-F1,6P as the internal standard. Means ±SEM from five independent experiments (n = 5) were graphed. Student’s t-test.
(TIF)

S1 Table Primers used in this study. (XLSX)

S2 Table Plasmids used in this study. (XLSX)

S3 Table Transgenic parasites used in this study. (XLSX)

S4 Table Abbreviations. (XLSX)

S5 Table The proteomics dataset used in Fig 6. (XLSX)

S6 Table The metabolomics dataset used in Fig 7A–7C. (XLSX)

S7 Table The metabolomics dataset used in Fig 7D. (XLSX)

S8 Table The metabolomics dataset used in Fig 7E. (XLSX)

S9 Table The metabolomics dataset used in Fig 7F. (XLSX)

S10 Table The metabolomics dataset used in S5 Fig. (XLSX)

S11 Table The raw mass spectral data related to Fig 7 and S5 Fig. (XLSX)

S12 Table The mass spectral data used in S8 Fig. (XLSX)
