# Supplementary figures and images for: Metabolic flexibilities and vulnerabilities in the pentose phosphate pathway of the zoonotic pathogen Toxoplasma gondii
Source: PLoS Pathog. 2022 Sep 19;18(9):e1010864. doi: 10.1371/journal.ppat.1010864 (PMC9521846; doi:10.1371/journal.ppat.1010864)

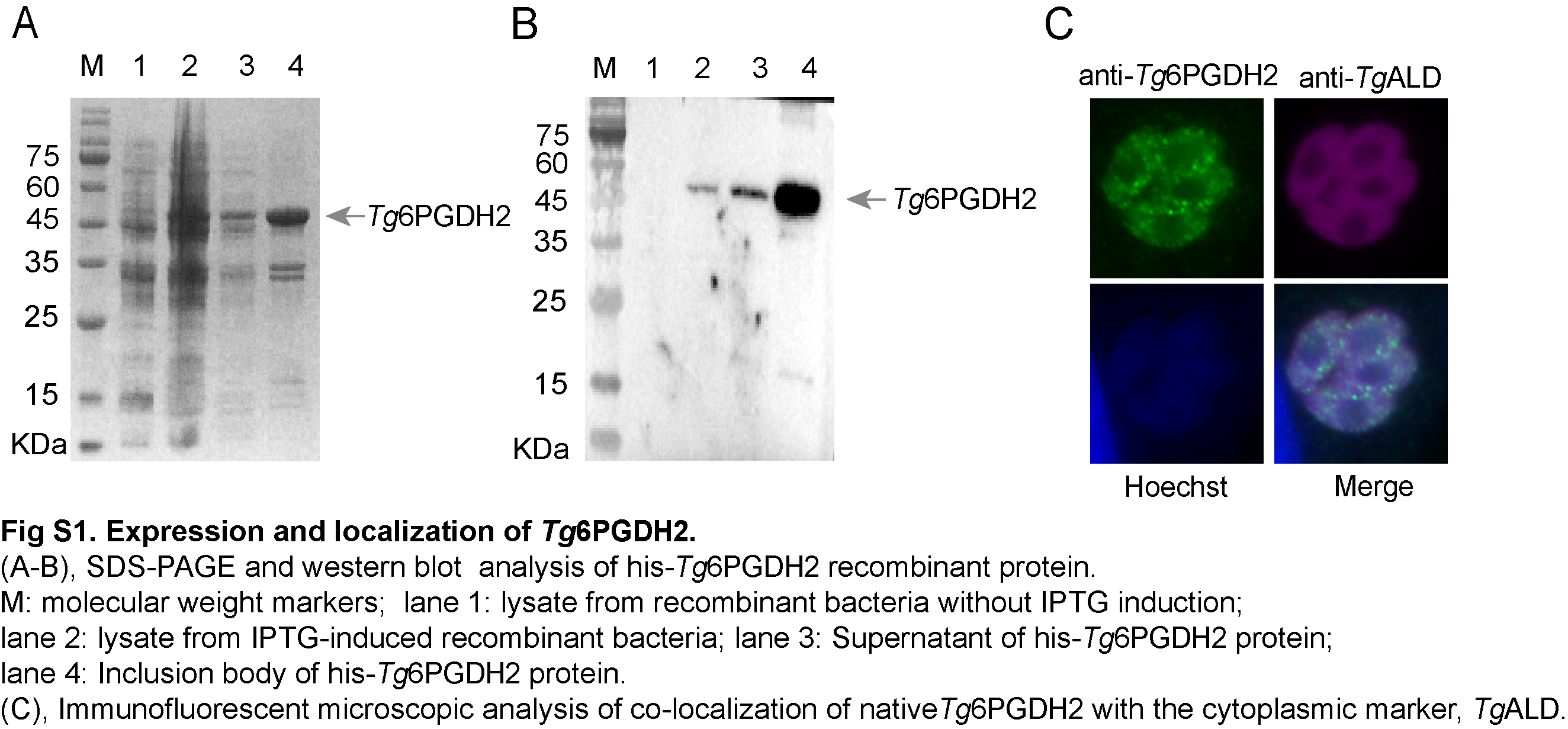

Supplement: S1 Fig — A-B, SDS-PAGE and western blot analysis of his-Tg6PGDH2 recombinant protein. M: molecular weight markers; lane 1: lysate from recombinant bacteria without IPTG induction; lane 2: lysate from IPTG-induced recombinant bacteria; lane 3: Supernatant of his-Tg6PGDH2 protein; lane 4: Inclusion body of his-Tg6PGDH2 protein. C, Immunofluorescent microscopic analysis of co-localization of native Tg6PGDH2 with the cytoplasmic marker, TgALD. (TIF) [file ppat.1010864.s001.tif]

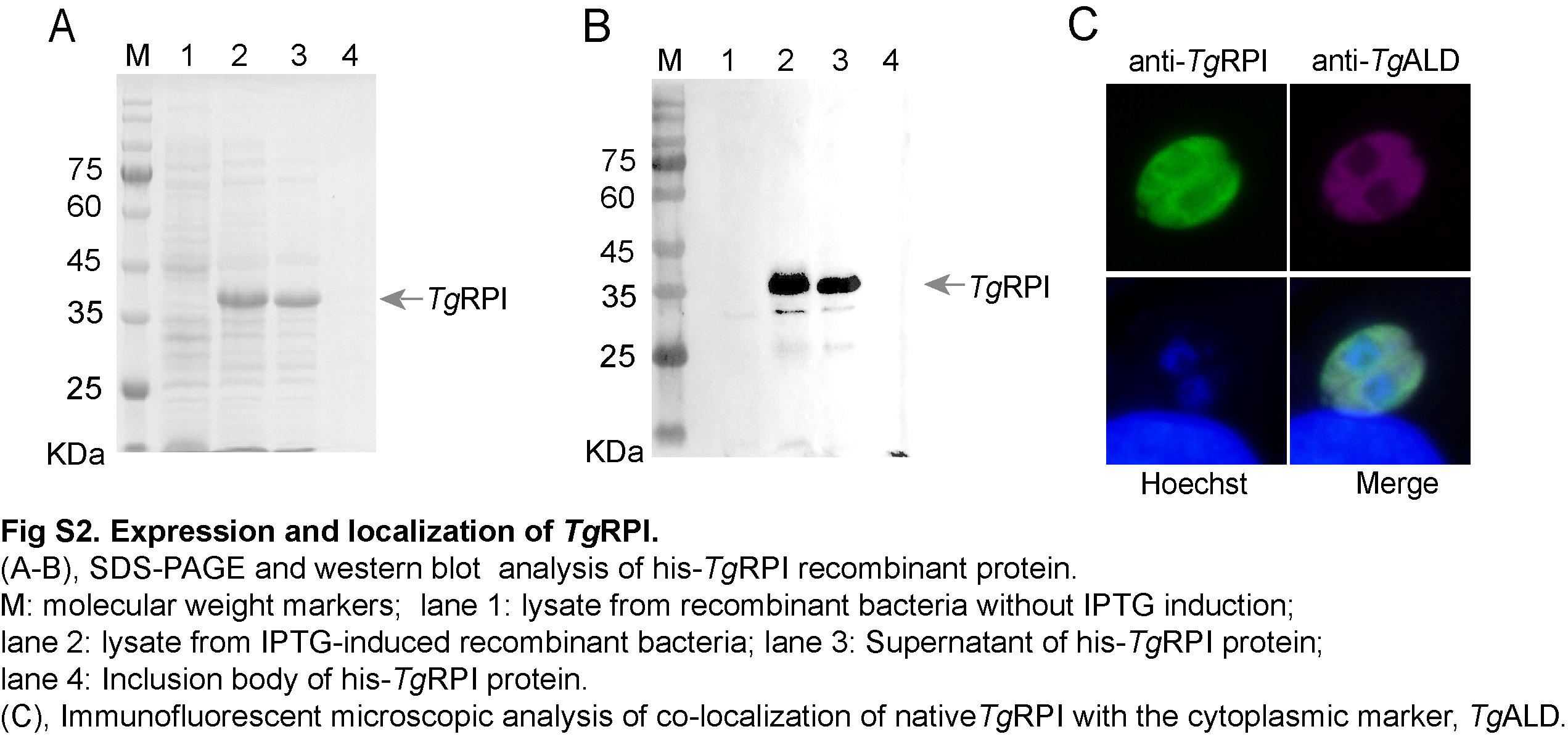

Supplement: S2 Fig — A-B, SDS-PAGE and western blot analysis of his-TgRPI recombinant protein. M: molecular weight markers; lane 1: lysate from recombinant bacteria without IPTG induction; lane 2: lysate from IPTG-induced recombinant bacteria; lane 3: Supernatant of his-TgRPI protein; lane 4: Inclusion body of his-TgRPI protein. C, Immunofluorescent microscopic analysis of co-localization of native TgRPI with the cytoplasmic marker, TgALD. (TIF) [file ppat.1010864.s002.tif]

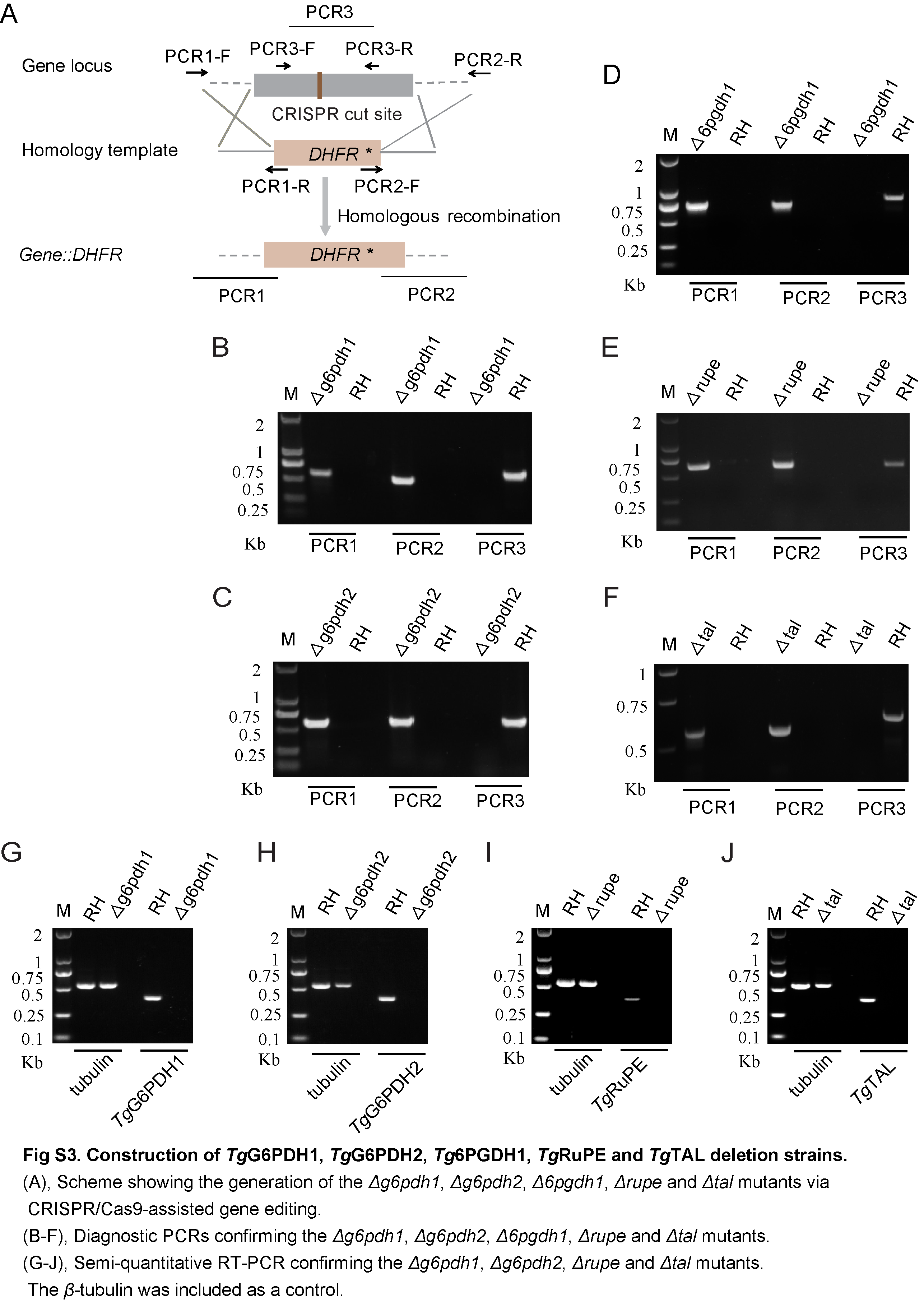

Supplement: S3 Fig — (A), Scheme showing the generation of the Δg6pdh1, Δg6pdh2, Δ6pgdh1, Δrupe and Δtal mutants via CRISPR/Cas9-assisted gene editing. (B-F), Diagnostic PCRs confirming the Δg6pdh1, Δg6pdh2, Δ6pgdh1, Δrupe and Δtal mutants. (G-J), Semi-quantitative RT-PCR confirming the Δg6pdh1, Δg6pdh2, Δrupe and Δtal mutants. The β-tubulin was included as a control. (TIF) [file ppat.1010864.s003.tif]

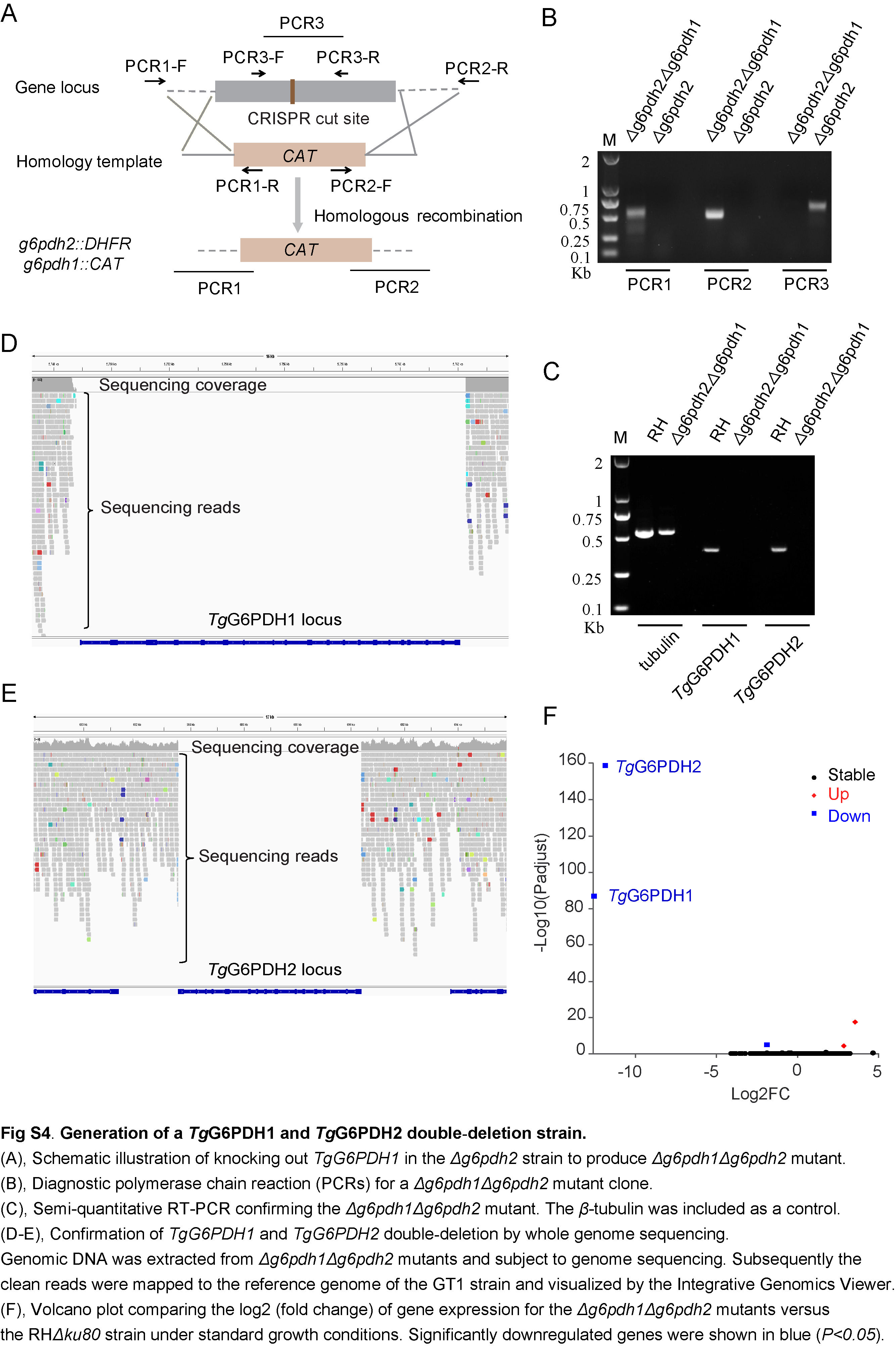

Supplement: S4 Fig — (A), Schematic illustration of knocking out TgG6PDH1 in the Δg6pdh2 strain to produce Δg6pdh1Δg6pdh2 mutant. (B), Diagnostic polymerase chain reaction (PCRs) for a Δg6pdh1Δg6pdh2 mutant clone. (C), Semi-quantitative RT-PCR confirming the Δg6pdh1Δg6pdh2 mutant. The β-tubulin was included as a control. (D-E), Confirmation of TgG6PDH1 and TgG6PDH2 double-deletion by whole genome sequencing. Genomic DNA was extracted from Δg6pdh1Δg6pdh2 mutants and subject to genome sequencing. Subsequently, the clean reads were mapped to the reference genome of the GT1 strain and visualized by the Integrative Genomics Viewer. (F), Volcano plot comparing the log2 (fold change) gene expression for the Δg6pdh1Δg6pdh2 mutants versus the RHΔku80 strain under standard growth conditions. Significantly downregulated genes were shown in blue (P<0.05). (TIF) [file ppat.1010864.s004.tif]

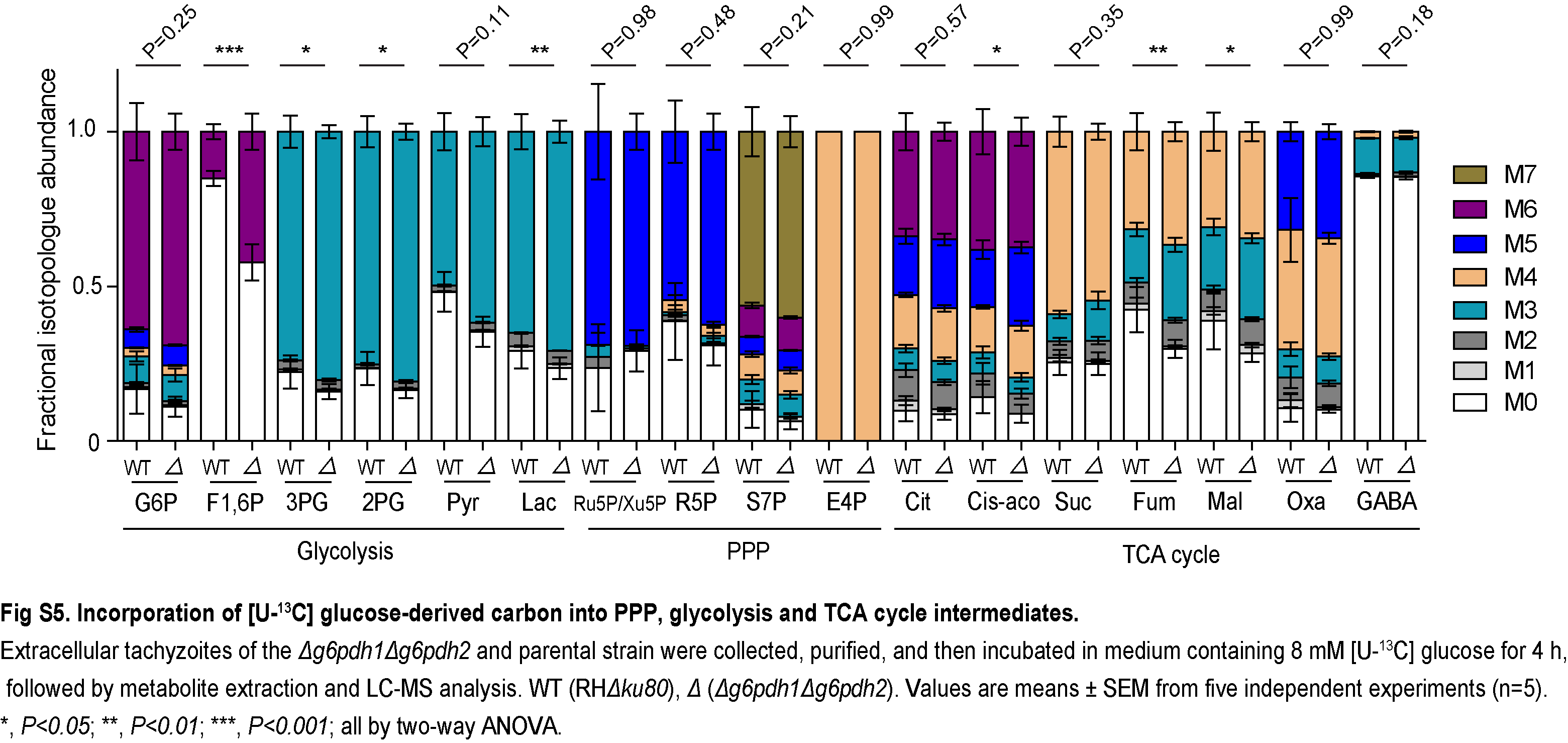

Supplement: S5 Fig — Extracellular tachyzoites of the Δg6pdh1Δg6pdh2 and parental strain were collected, purified, and then incubated in a medium containing 8 mM [U-13C] glucose for 4 h, followed by metabolite extraction and LC-MS analysis. WT (RHΔku80), Δ (Δg6pdh1Δg6pdh2). Values are means ± SEM from five independent experiments (n = 5). *, P<0.05; **, P<0.01; ***, P < .001; all by two-way ANOVA. (TIF) [file ppat.1010864.s005.tif]

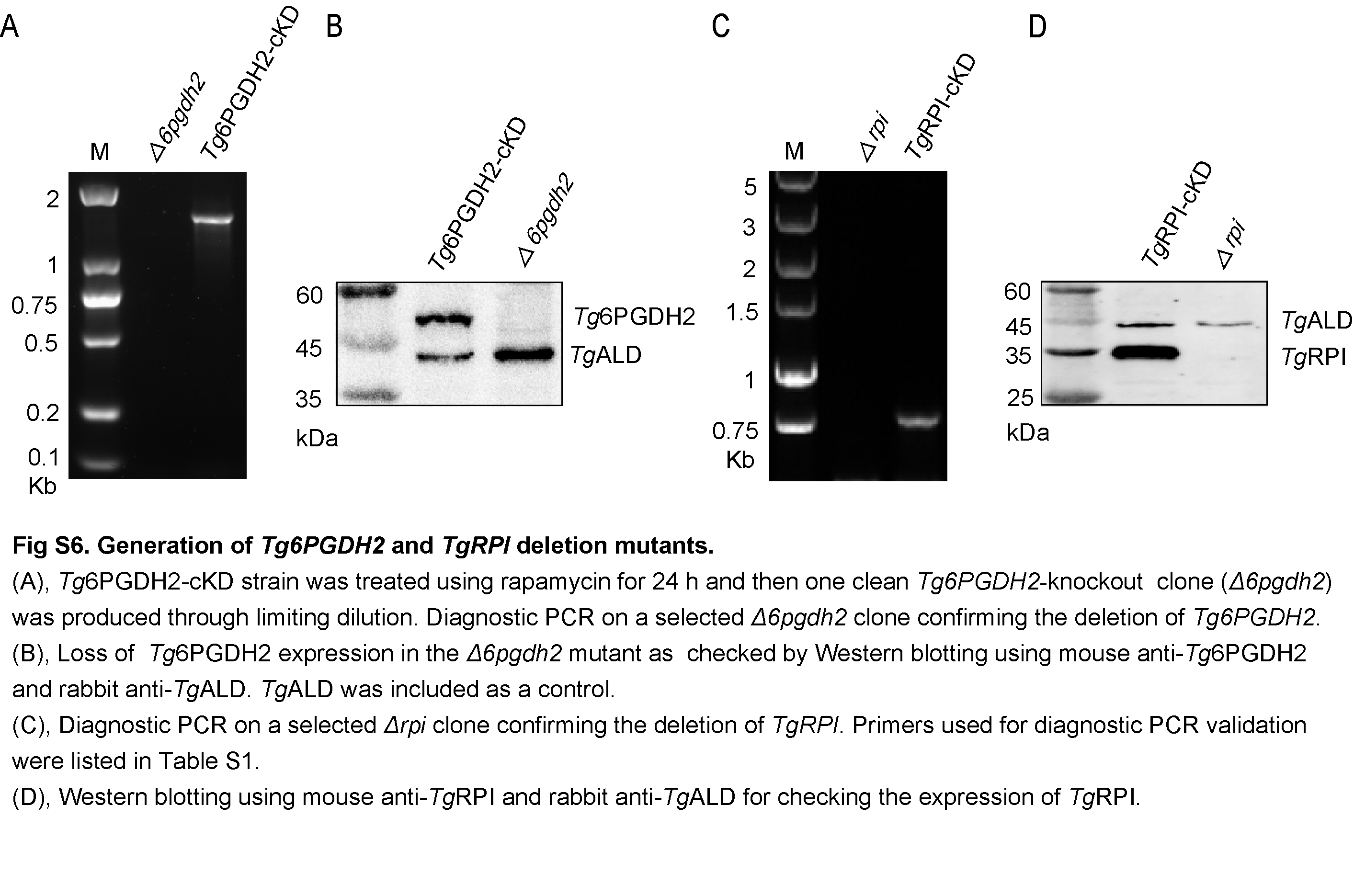

Supplement: S6 Fig — (A), Tg6PGDH2-cKD strain was treated using rapamycin for 24 h and then one clean Tg6PGDH2-knockout clone (Δ6pgdh2) was produced through limiting dilution. Diagnostic PCR on a selected Δ6pgdh2 clone confirming the deletion of Tg6PGDH2. (B), Loss of Tg6PGDH2 expression in the Δ6pgdh2 mutant as checked by Western blotting using mouse anti-Tg6PGDH2 and rabbit anti-TgALD. TgALD was included as a loading control. (C), Diagnostic PCR on a selected Δrpi clone confirming the deletion of TgRPI. Primers used for diagnostic PCR validation were listed in S1 Table. (D), Western blotting using mouse anti-TgRPI and rabbit anti-TgALD for checking the expression of TgRPI. (TIF) [file ppat.1010864.s006.tif]

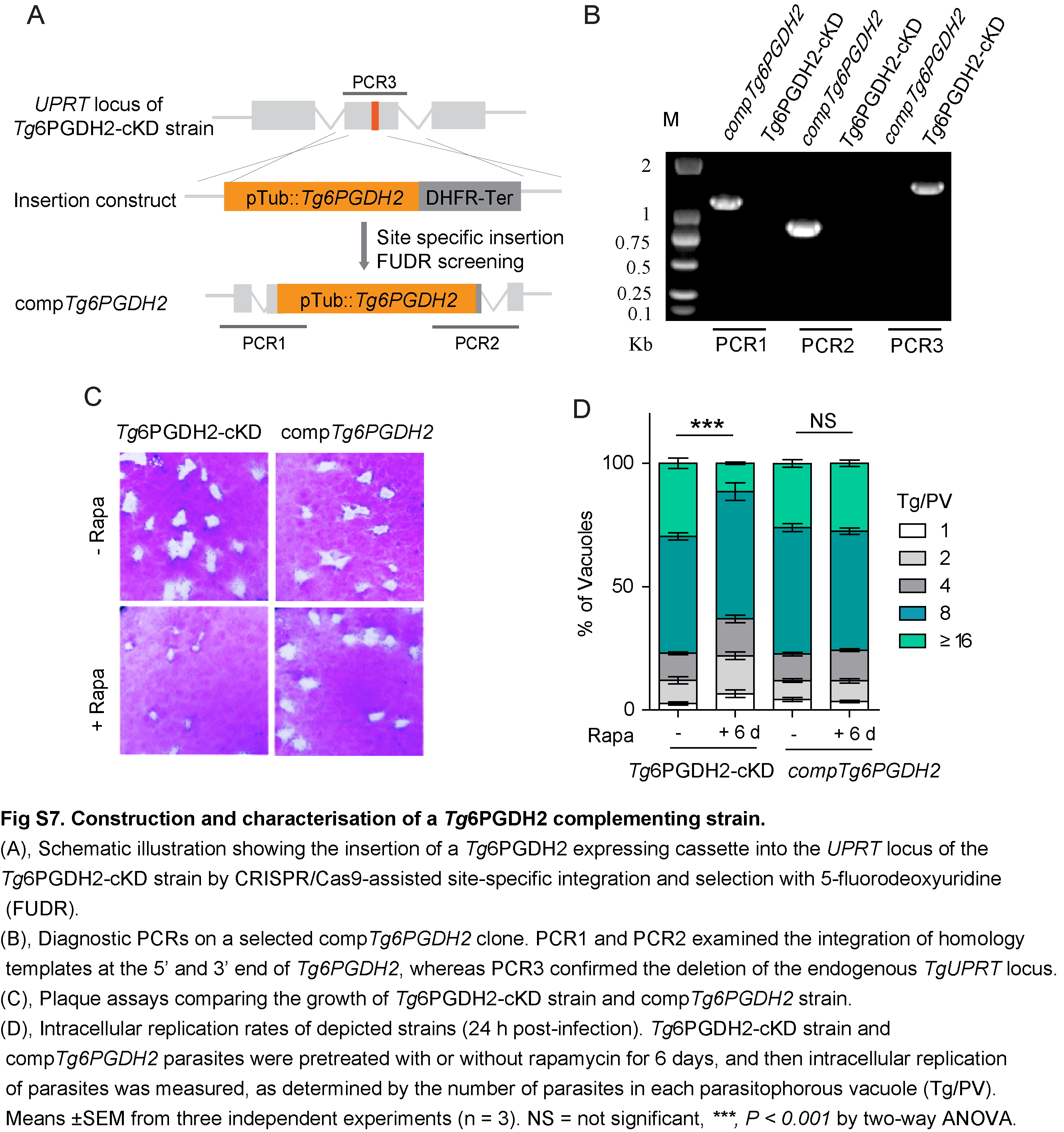

Supplement: S7 Fig — (A), Schematic illustration showing the insertion of a Tg6PGDH2 expressing cassette into the UPRT locus of the Tg6PGDH2-cKD strain by CRISPR/Cas9-assisted site-specific integration and selection with 5-fluorodeoxyuridine (FUDR). (B), Diagnostic PCRs on a selected compTg6PGDH2 clone. PCR1 and PCR2 examined the integration of homology templates at the 5’ and 3’ end of Tg6PGDH2, whereas PCR3 confirmed the deletion of the endogenous UPRT locus. (C), Plaque assays comparing the growth of Tg6PGDH2-cKD strain and compTg6PGDH2 strain. (D), Intracellular replication rates of depicted strains (24 h post-infection). Tg6PGDH2-cKD strain and compTg6PGDH2 parasites were pretreated with or without rapamycin for 6 days, and then intracellular replication of parasites was measured, as determined by the number of parasites in each parasitophorous vacuole (Tg/PV). Means ±SEM from three independent experiments (n = 3). NS = not significant, ***, P < 0.001 by two-way ANOVA. (TIF) [file ppat.1010864.s007.tif]

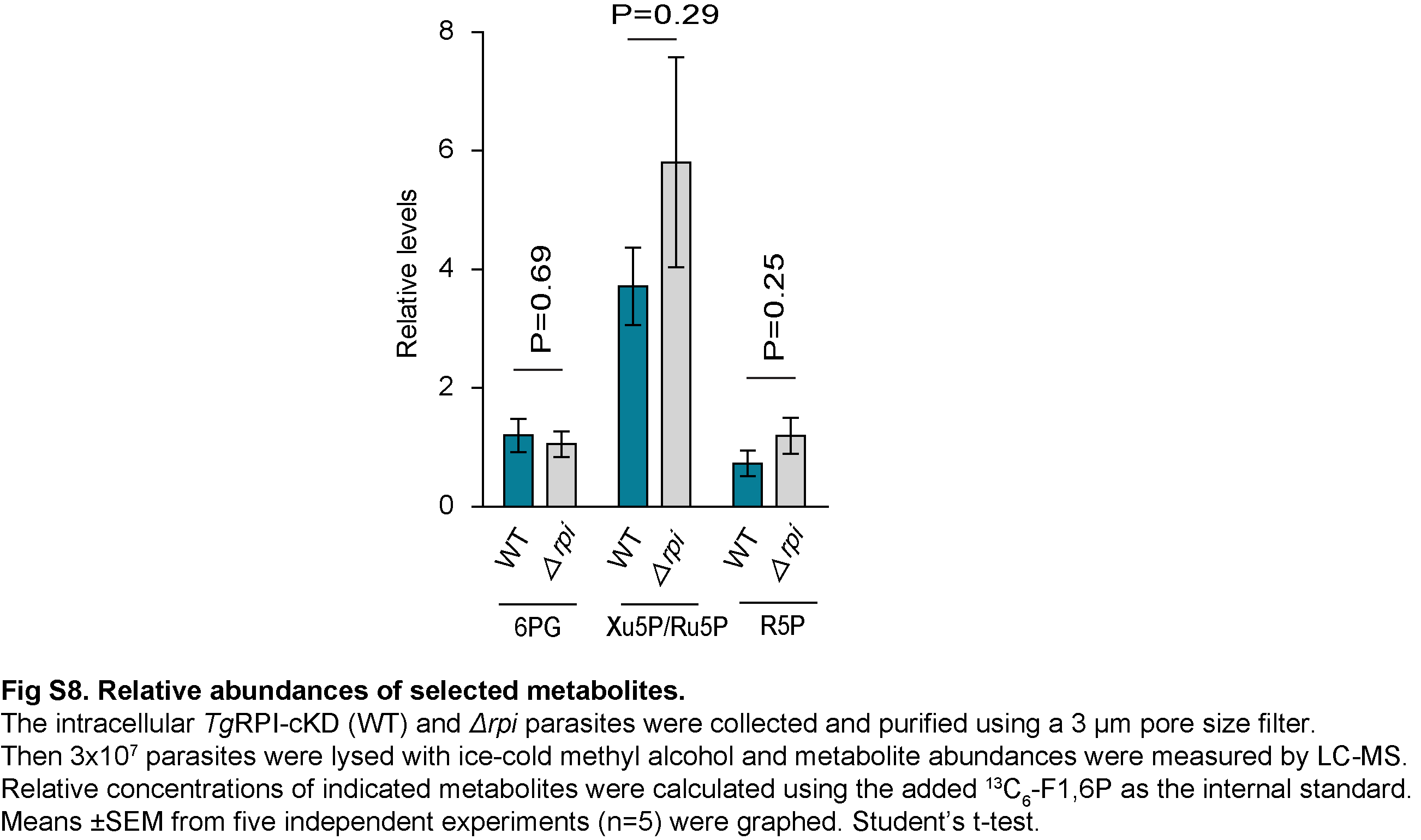

Supplement: S8 Fig — The intracellular TgRPI-cKD (WT) and Δrpi parasites were collected and purified using a 3 μm pore size filter. Then 3x10^7^ parasites were lysed with ice-cold methyl alcohol and metabolite abundances were measured by LC-MS. Relative concentrations of indicated metabolites were calculated using the added 13C6-F1,6P as the internal standard. Means ±SEM from five independent experiments (n = 5) were graphed. Student’s t-test. (TIF) [file ppat.1010864.s008.tif]
